# Quantitative profiling of oxylipins through comprehensive LC-MS/MS analysis: application in cardiac surgery

**DOI:** 10.1007/s00216-012-6226-x

**Published:** 2012-07-20

**Authors:** Katrin Strassburg, Annemarie M. L. Huijbrechts, Kirsten A. Kortekaas, Jan H. Lindeman, Theresa L. Pedersen, Adrie Dane, Ruud Berger, Arjan Brenkman, Thomas Hankemeier, John van Duynhoven, Eric Kalkhoven, John W. Newman, Rob J. Vreeken

**Affiliations:** 1 Leiden Amsterdam Centre for Drug Research, Leiden University, Einsteinweg 55, 2300 RA Leiden, The Netherlands; 2 Department of Metabolic and Endocrine Diseases, University Medical Centre Utrecht, Utrecht, The Netherlands; 3 Netherlands Metabolomics Centre, Einsteinweg 55, 2300 RA Leiden, The Netherlands; 4 Department of Cardiothoracic Surgery, Leiden University Medical Center, Albinusdreef 2-Dialyse BO-P, 2333 ZA Leiden, The Netherlands; 5 Department of General Surgery, Leiden University Medical Center, Leiden, The Netherlands; 6 USDA-ARS Western Human Nutrition Research Center, 430 West Health Sciences, Davis, CA USA; 7 Department of Nutrition, University of California, 430 West Health Sciences, Davis, CA USA; 8 Unilever Research and Development, Olivier van Noortlaan 120, 3133 AT Vlaardingen, The Netherlands; 9 Laboratory of Biophysics, Wageningen University, Dreijenlaan 3, 6703 HA Wageningen, The Netherlands; 10 Department of Analytical BioSciences, Leiden Amsterdam Centre for Drug Research, Leiden University, Einsteinweg 55, 2300 RA Leiden, The Netherlands

**Keywords:** HPLC-MS/MS, dMRM, 12-HETE, 12-HEPE

## Abstract

Oxylipins, including eicosanoids, affect a broad range of biological processes, such as the initiation and resolution of inflammation. These compounds, also referred to as lipid mediators, are (non-) enzymatically generated by oxidation of polyunsaturated fatty acids such as arachidonic acid (AA). A plethora of lipid mediators exist which makes the development of generic analytical methods challenging. Here we developed a robust and sensitive targeted analysis platform for oxylipins and applied it in a biological setting, using high performance liquid chromatography coupled to tandem mass spectrometry (HPLC-MS/MS) operated in dynamic multiple reaction monitoring (dMRM). Besides the well-described AA metabolites, oxylipins derived from linoleic acid, dihomo-γ-linolenic acid, α-linolenic acid, eicosapentaenoic acid and docosahexaenoic acid were included. Our comprehensive platform allows the quantitative evaluation of approximately 100 oxylipins down to low nanomolar levels. Applicability of the analytical platform was demonstrated by analyzing plasma samples of patients undergoing cardiac surgery. Altered levels of some of the oxylipins, especially in certain monohydroxy fatty acids such as 12-HETE and 12-HEPE, were observed in samples collected before and 24 h after cardiac surgery. These findings indicate that this generic oxylipin profiling platform can be applied broadly to study these highly bioactive compounds in relation to human disease.

**Electronic supplementary material:**

The online version of this article (doi:10.1007/s00216-012-6226-x) contains supplementary material, which is available to authorized users.

**Figure:**
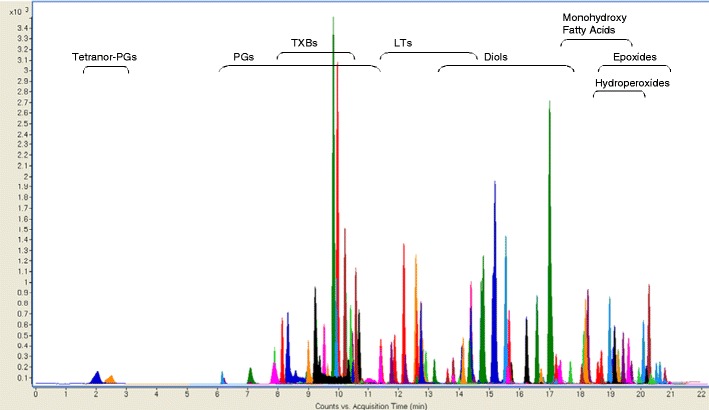
LC-MS/MS chromatogram of 104 oxylipins on a Triple Quadrupole MS system employing dynamic MRM in the Negative Ion Electrospray mode

## Introduction

Oxylipins are oxygenated metabolites derived from poly-unsaturated fatty acids (PUFAs) such as arachidonic acid (AA), linoleic acid (LA), eicosapentaenoic acid (EPA), docosahexaenoic acid (DHA), and dihomo-γ-linolenic acid (DGLA). The oxygenated 20-carbon products, collectively referred to as eicosanoids, are the best-described lipid mediators. Among their many functions, these lipids are potent inflammatory modulators and thus have been associated with responses to cardiovascular diseases, host defense, tissue injury, and surgical intervention [1–3].

**Fig. 1:**
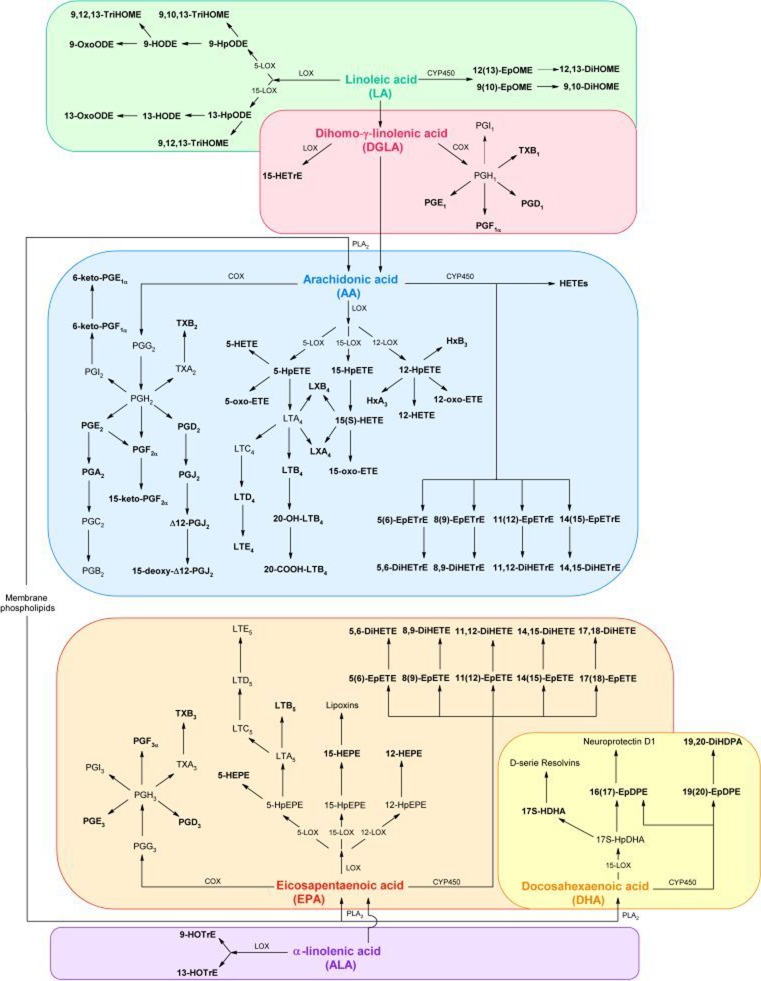
Biosynthesis of oxylipins derived from linoleic acid (LA; *green*), dihomo-γ-linolenic acid (DGLA; *red*), arachidonic acid (AA; *blue*), eicosapentaenoic acid (EPA; *orange*), docosahexaenoic acid (DHA; *yellow*), and α-linolenic acid (ALA; *purple*) via lipoxygenase (LOX), cyclooxygenase (COX), and cytochrome P450 (CYP450) pathways; PLA_2_: phospholipase A_2_; metabolites (*bold marked*) are included in the oxylipin library of the described analytical platform

Through both, enzymatic and non-enzymatic oxidation processes, these PUFAs are transformed into a variety of oxylipins. They can be generated from the different precursor PUFAs by three main classes of enzymes—cyclooxygenase (COX), lipoxygenase (LOX), and cytochrome P450 (CYP450)—and thus form a complex pool of bioactive components (Fig. 1). Prostaglandins (PG) and thromboxanes (TX) are generated via the initial oxidation through COX pathways [4]. Prostanoids derived from AA are generally known as class 2 PGs and TXs due to their residual double-bond number, e.g., PGE_2_ and TXB_2_. Class 1 and 3 PGs and TX are similarly produced through the oxidation of DGLA and EPA, respectively [5]. Leukotrienes, likewise involved in pro-inflammatory signaling, especially in chemotaxis of leukocytes, are generated via the LOX pathways [4, 6]. In mammalian cells, three LOX hydroperoxidases families exist, 5-LOX, 15-LOX, and 12-LOX, from which 5-LOX generates leukotrienes. Other LOX-dependent products include the mid-chain alcohols such as the AA-derived hydroxyeicosatetraenoic acids (HETEs), as well as the LA-derived hydroxyoctadecadienoic acids (HODEs) and EPA-derived hydroxypentaenoic acids (HEPEs). HETEs are known to be bioactive lipid mediators in chemotaxis and degranulation processes of neutrophils [7–9]. Omega-terminal oxidation of these PUFAs by CYP450 yields additional metabolites such as epoxides, diols, and further HETEs. Epoxides derived from AA are known to be associated to cardiovascular functions, in particular acting as vasodilators of human coronary arteries [10, 11]. For epoxides derived from EPA, similar functions are evident [12]. While conversion of the epoxy fatty acids to diols reduces their vasoactive properties, the diols themselves appear to have distinct functions, for instance as ligands of the peroxisome proliferator activated receptor alpha subtype (PPARα) [13, 14].

Although several oxylipins have been allocated to regulatory functions, the biological impact for many others remains unclear. Therefore, the bioactive potential of the less well-characterized lipids, either as unique functions or interaction with structural analogs from different precursor PUFAs, is likely to provide a rich source of novel insights into the regulation of mammalian responses to disease and tissue injury. Covering a wide range of these highly bioactive compounds still remains a challenge with modern analytical techniques. As a common approach, immunoassays are performed, either as radioimmunoassay [15, 16], enzyme immunoassays [17], or luminescent immunoassays [18, 19]. They are specific and focus on one or only few compounds. Gas chromatography coupled to mass spectrometry (GC-MS) technology provides a sensitive methodology for a broad range of oxylipins [20]. However, to increase volatility, derivatization steps are essential. To avoid additional modifications, liquid chromatography-mass spectrometric (LC-MS) techniques are one of the most sensitive and specific tools to simultaneously study many analogous analytes. Numerous LC-MS/MS methods have been described in literature for identification and quantification of low abundant oxylipins using multiple reaction monitoring (MRM) as an ion selective mode. Many methods focus on certain component classes of oxylipins as for instance prostaglandins [21–23], non-prostanoid eicosanoids [24], COX and LOX derived eicosanoids [25, 26], CYP450 related compounds [27, 28], and AA derived lipid mediators including metabolites of all three pathways [29–31]. However, the bioactive lipids of different origins are produced within the same cascade and cross-linked in a complex regulatory network (Fig. 1). Thus, the complex combination of chemically and structurally related oxylipins derived from the different PUFAs has become a challenge in modern LC-MS/MS techniques [32–36]. The development of a generic analytical oxylipin platform, intended for the application to a variety of biological matrices, will provide valuable information on metabolites derived from different precursor PUFAs and contribute importantly to our knowledge on inflammation processes.

Within this work we expand these methods to include an even broader array of prostanoids while demonstrating the utility of dynamic multiple reaction monitoring (dMRM) mode to focus on short retention time windows and enhance sensitivity. We applied our platform to human plasma derived from patients a day before and 24 h after cardiac surgery. Previous studies have demonstrated involvement of eicosanoids in myocardial ischemia/reperfusion injury. Arachidonic acid accumulates in the reperfused heart after an ischemic period and levels of prominent eicosanoids like PGE_2_, TXB_2_, and 6-keto-PGF_1α_ change rapidly after reperfusion [37–39]. In this study, we focus on late phase (i.e., 24 h post surgery) changes in circulating eicosanoids and further demonstrate the applicability of this generic LC-MS/MS platform to monitor physiological levels of a broad range of eicosanoids and related metabolites derived from different PUFAs in human plasma.

## Material and methods

### Materials

Ultra performance liquid chromatography (UPLC)-grade acetonitrile, isopropanol, methanol, ethyl-acetate, and water were purchased from Biosolve (the Netherlands). Glacial acetic acid was from Sigma-Aldrich (St. Louis, MO). High performance liquid chromatography (HPLC) was performed with the Ascentis Express C18 (2.1 × 150 mm, 2.7 μm) column from Sigma-Aldrich (St. Louis, MO). Solid phase extraction (SPE) was accomplished with Oasis HLB (60 mg/30 μm) cartridges from Waters (Milford, MA). Deuterated and non-deuterated oxylipin standards were purchased either from Cayman Chemicals (Ann Arbor, MI), Biomol (Plymouth Meeting, PA), or Larodan (Malmö, Sweden). Human EDTA-plasma for method validation was provided by Richmond Pharmacology Ltd. (London, UK).

### Patient population

Oxylipin profiling was performed on EDTA-plasma derived from patients before and after cardiac surgery. The study protocols were approved by the local ethics committee, and informed consent was obtained from each patient. All patients were male and with good left ventricular function and 55 (±5.4) years of age with a BMI of 27.1 (±4.1) kg/m^2^. As patients develop a state of inflammation upon cardiac surgery, these patients provided a well controlled sample set to monitor levels of oxylipins in their function as bioactive lipid mediators after cardiac surgery. During cardiac arrest (myocardial ischemia), cardiac surgery was executed with use of a heart–lung machine. After 124.2 (±21.9) min of ischemia, the aortic cross-clamp was removed to restore cardiac blood flow (i.e., reperfusion).

To avoid any effects of heparin, such as increased levels of oxylipins by heparin induced phopholipase A2 activity, described in literature [40, 41], samples collected during the period in which heparin was administered to the patient were not used. Heparin administrated in conjunction with heart–lung machine use is neutralized by protamine sulfate, administrated in the operating room directly after the surgical intervention. Based on our own confirmation of a heparin-dependent elevation in circulating oxylipins (data not shown) and their response to protamine sulfate treatment, we chose to analyze samples taken the day before surgery (no heparin administrated to the patient) and 24 h after reperfusion of the heart, when no influence of heparin could be detected. All baseline and 24 h post-reperfusion samples were taken at the same moment during the day, between 12:00 noon and 2:00 p.m., to reduce the potential impact of circadian rhythm-dependent cycling which can affect several oxylipins. While they show a morning peak, the concentrations reduce during the day [42–44].

### Oxylipin extraction from human blood plasma

The samples (10 mL) were taken the day before surgery by venipuncture (baseline) from the brachial vein and 24 h after reperfusion from the radial artery. All samples were collected in pre-cooled tubes containing EDTA (BD Vacutainer, Plymouth, UK) and placed on melting ice immediately. Blood samples were centrifuged (1.550×*g*, 10 min, 4 °C) and the derived plasma supernatant was re-centrifuged (10.000×*g*, 4 min, 4 °C) to obtain leukocyte and thrombocyte poor plasma. Aliquots were stored at −80 °C until extraction. The extraction was performed as described previously [36]. For oxylipin analysis 250 μL aliquots were taken. After thawing on ice, the samples were treated immediately with antioxidants (0.2 mg BHT/EDTA) and spiked with 5 μL of internal standards (ISTDs) of a concentration of 1,000 nM, resulting in 100 nM after reconstitution which is within the linear range of the method. Compound extraction was performed with solid phase extraction using Oasis HLB (60 mg/30 μm). Oxylipins were eluted with 2 mL ethyl acetate after wetting the cartridge with 0.5 mL methanol. In contrast to Shearer and coauthors [36], the eluent was reduced under nitrogen stream instead under vacuum. The dried extract was subsequently reconstituted in 50 μL solution of methanol and acetonitrile (1:1) containing 100 nM 1-cyclohexyluriedo-3-dodecanoic acid (CUDA) as a quality marker for the analysis. Afterwards, the extract was filtered by centrifugation using Amicon Ultrafree-MC Durapore PVDF filter (pore-size 0.1 μM; Millipore, Bedford, MA).

### Liquid chromatography and mass spectrometry

Samples were analyzed by liquid chromatography (Agilent 1260, San Jose, CA, USA) coupled to electrospray ionization on a triple quadrupole mass spectrometer (Agilent 6460, San Jose, CA, USA). For analysis 5 μL of the extract was injected. The auto sampler was cooled at 10 °C. Chromatographic separation was achieved on an Ascentis Express (2.1 × 150 mm, 2.7 μm particles; Sigma-Aldrich Supelco) column using a flow rate of 0.35 mL/min at 40 °C during a 26 min gradient (0–3.5 min from 15 % B to 33 % B, 3.5–5.5 min B to 38 %, 5–7 min to 42 % B, 7–9 min to 48 % B, 9–15 min to 65 % B, 15–17 min to 75 % B, 17–18.5 min to 85 % B, 18.5–19.5 min to 95 % B, from 19.5 to 21 min to 15 % B, 21–26 min 15 % B), while using the solvents A, 0.1 % acetic acid, and B, 90:10 *v*/*v* acetonitril/isopropanol. Electrospray ionization was performed in the negative ion mode using N_2_ at a pressure of 35 psi for the nebulizer with a flow of 10 L/min and a temperature of 300 °C, respectively. The sheath gas temperature was 350 °C with a flow rate of 11 L/min. The capillary was set at 3,500 V and the nozzle voltage was 1,000 V.

To detect the individual oxylipins, MRM in negative ion mode was performed with individually optimized fragmentor voltage and collision energies (Optimizer application, MassHunter, Agilent). MRM transitions were achieved by flow injection of pure standards and the optimizer application and were compared to literature when available for the certain compounds. The detailed list of MRM transitions can be found in the Electronic Supplementary Material Table S-1. Instead of defining certain time segments, with fixed dwell times per compound, dynamic MRM was used, assuring optimal dwell time and sufficient data points per peak.

### Data preprocessing

Peak determination and peak area integration was performed with Mass Hunter Quan (Agilent, Version B.04.00) while auto-integration was manually inspected and corrected if necessary. The obtained peak areas of targets were corrected by appropriate internal standards (ISTD) and calculated response ratios were used throughout the analysis.

### Method validation

The method validation was performed as in-house 3-day protocol to determine linearity, LOD and LOQ, inter- and intra-day variation, reproducibility, matrix effect, and recovery for all compounds. Compound stability was not included in this validation, but is being performed in a dedicated experiment and currently ongoing (stability period up to 48 weeks at −80 °C without antioxidants). Preliminary data on this stability experiment showed no significant degradation over a period of 6 month (data not shown). We spiked pooled EDTA-plasma (Richmond Pharmacology, London, UK) with the purchased standard compounds in different concentrations varying from a minimum of 2 nM up to 558 nM in 8 levels (corresponding to levels of 3 to 1,260 pg; Electronic Supplementary Material Table S-2) to determine the linearity. Each calibration level was extracted twice and injected three times. Calibration curves were calculated by linear regression with 1/S^2^ weighing. Not all levels were included for each analyte. Only if a response was measured, i.e., for many analytes the low levels were not included. Furthermore, if the endogenous concentration was relatively high compared to the additionally spiked concentration, there is no difference in measured response when going to the next spiked level. For the calibration line, only levels were included if a significant response increase occurred from level to level. The responses were compared by means of *t* tests going from low to high level. For example, if the tests show no significant differences between level C0 compared to level C1 and C1 versus C2 but a significantly higher response for C3 compared to C2 than the measurements for C0 and C1 were not included in the calibration line and C2 was the lowest included level. LOD and LOQ were determined by signal to noise ratio (S/N) higher 3 and 10, respectively. For the calculation of possible batch-to-batch effect, we compared three different concentrations, lower level (C3), middle (C5), and high level (C7). Three different batches were prepared at three following days. To determine the recovery of the oxylipins, we spiked the pooled plasma with 11 deuterated ISTDs prior to the extraction step and in parallel after the last step of the extraction procedure. The labeled standards are non-endogenous and can be distinguished from endogenous ones with mass spectrometry. The 11 chosen ISTDs were considered to be representative for the different compound classes. Recovery was calculated as Area_spiked before_/Area_spiked after._

## Results and discussion

**Fig. 2:**
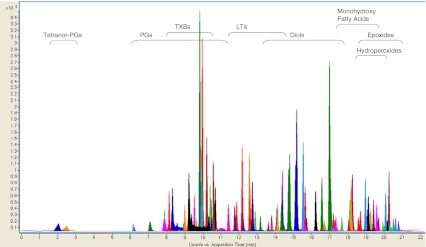
LC-MS/MS chromatogram of 104 oxylipins performed on a triple quadrupole employing dynamic MRM in negative mode (exp. details; see the “Materials and methods” section and Table 2)

A robust and sensitive quantitative method for profiling oxylipins such as eicosanoids and related lipid mediators biosynthesized from AA and other PUFAs was developed. As the majority of these compounds are present in plasma at low concentrations, their detection and quantification require methods with high sensitivity. Our LC-MS/MS method, employing dynamic MRM (dMRM), allows the evaluation of more than 100 oxylipins in a targeted approach (Fig. 2). The performance of dMRM allows the triple quadrupole system to be focused directly on the expected analyte retention time (*t*_R_) with a defined detection window instead of user-defined time segments to capture groups of closely eluting compounds. Allowing a constant cycle time for each transition, the dMRM procedure improves peak symmetry. Moreover, since transitions are only monitored in the defined detection windows in contrast to the whole time period of a standard MRM time segment, the rate of false positive detection (i.e., peak misidentification) is diminished. This is especially relevant for oxylipin families, which encompasses many isomeric and isobaric compounds. Furthermore, sensitivity can be enhanced since optimal dwell times can be achieved by reducing overlapping ion transitions [45].

**Fig. 3:**
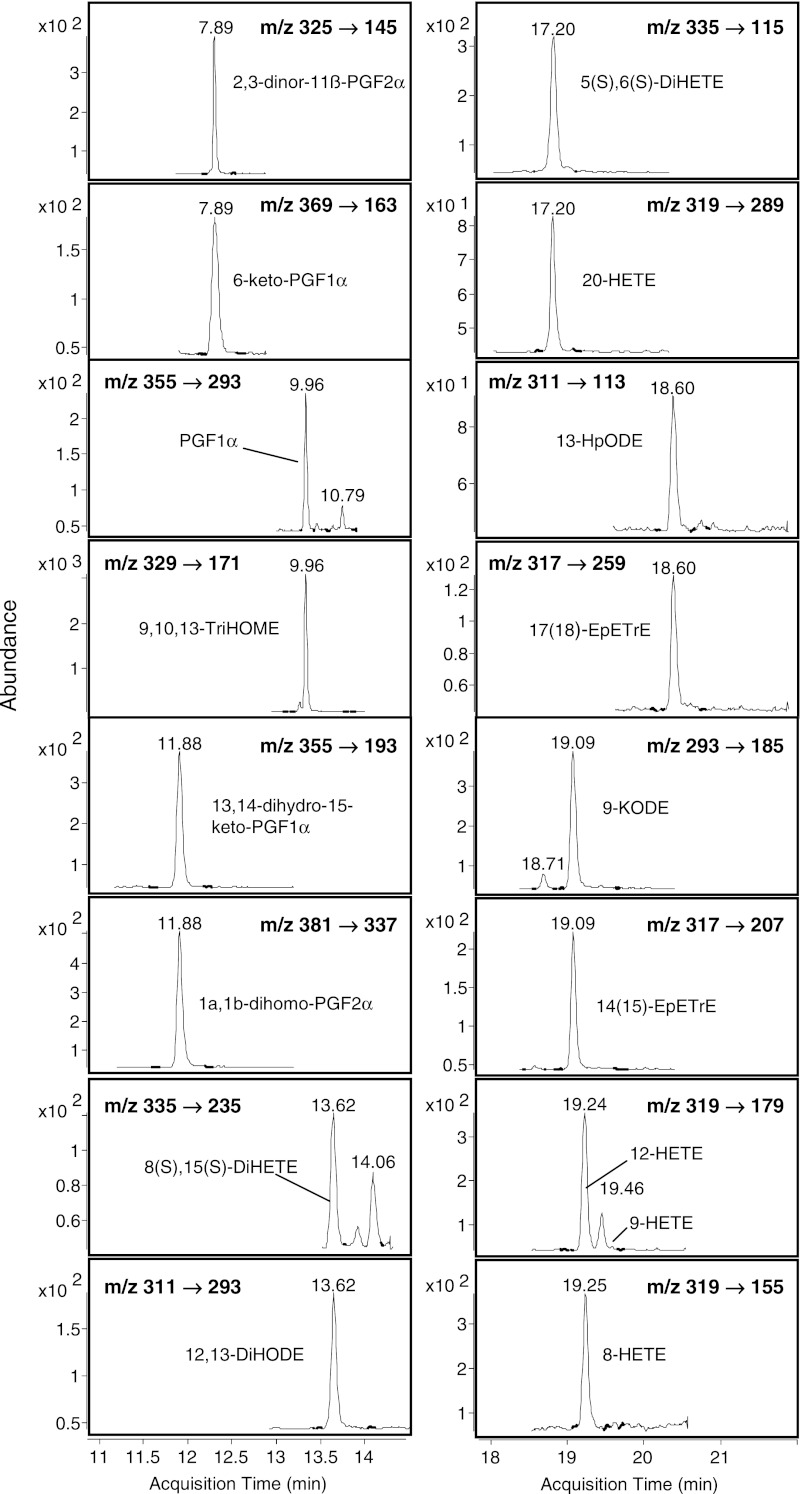
LC-MS/MS extracted chromatograms of co-eluting oxylipins acquired by dynamic MRM. Retention time and specific MRM transitions are shown for each oxylipin

For each compound, optimal transitions were determined to assure optimal peak detection (Electronic Supplementary Material Fig. S-1 and Table S-1). In the case of co-eluting metabolites, compound specific precursor ions and their corresponding fragment ions allowed separation for selective detection and quantification of those compounds (Fig. 3). For instance, for 2,3-dinor-11ß-PGF_2α_ (*m*/*z* 325 → 145) and 6-keto-PGF_1α_ (*m*/*z* 369 → 163), both elute at *t*_R_ 7.89 min. Additional pairs were PGF_1α_/9,10,13-TriHOME; 13,14-dihydro-15-keto-PGF_1α_/1a,1b-dihomo-PGF_2α_; 8,15-DiHETE/12,13-DiHODE; 5,6-DiHETE/20-HETE; 13-HpODE/17(18)-EpETE; and 9-KODE/14(15)-EpETE (Fig. 3). For co-eluting isobars a unique fragment ion was chosen, as for 12-HETE (*m*/*z* 319 → 179) and 8-HETE (*m*/*z* 319 → 115), both eluting at *t*_R_ 19.24 (Fig. 3). However, some critical pairs could not be separated spectrally with the current method. Those compounds were 19-hydroxy-PGE_2_/20-hydroxy-PGE_2_, 19-hydroxy-PGF_2α_/20-hydroxy-PGF_2α_, as well as LTB_4_/12-epi LTB_4_ and 6-trans-LTB_4_/6-trans-12-epi LTB_4_. For detection and data evaluation, these compounds are therefore reported as combined amounts. For the hydroperoxides (HpETEs and HpODEs), we perceived a loss of an H_2_O molecule during ionization leading to [M–H–H_2_O]^−^. The water loss, especially for the hydroperoxides from AA, was also described previously [46].

Taken together, by employing dMRM and the detection of specified transitions in our HPLC-MS/MS approach, we were able to detect more than 100 different oxylipins.

### Method validation

Validation measurements were performed using pooled EDTA-plasma as a sample matrix. Linearity, sensitivity (LOD and LOQ), reproducibility, matrix effect, and recovery were determined (Table 1 and Electronic Supplementary Material Table S-3).

**Table 1:** Validation results of, in study samples, detected oxylipins

Compound name	Retention time	*R* ^2^	LOD [nM]	LOQ [nM]	Intra-batch effect RSD [%]	Batch-to-batch effect RSD [%]
Arachidonic acid						
TXB2	9.24	0.995	0.3	1.0	<7	13–22
PGF2α	9.96	0.998	0.9	2.9	<10	4–15
PGE2	10.23	0.999	0.2	0.6	<13	4–20
11β-PGE2	10.40	0.998	0.9	3.1	<8	3–19
13,14-dihydro PGF2α	10.79	0.990	11.4	38.2	<8	12–26
14,15-DiHETrE	15.65	0.997	0.3	1.0	<6	2–21
11,12-DiHETrE	16.23	0.993	0.5	1.7	<12	6–21
8,9- DiHETrE	16.71	0.993	0.3	1.0	<15	10–17
5,6-DiHETrE	17.34	0.994	0.4	1.2	<9	7–21
15-HETE	18.58	0.982	0.2	0.8	<11	6–18
11-HETE	18.97	0.987	0.3	0.9	<9	4–21
12-HETE	19.24	0.986	0.2	0.6	<9	4–17
8-HETE	19.25	0.990	1.4	4.7	<8	8–21
5-HETE	19.69	0.988	0.2	0.6	<11	14–20

Linoleic acid						
9,12,13-TriHOME	9.83	0.946	0.2	0.8	<32	9–16
9,10,13-TriHOME	9.96	0.953	0.5	1.8	<10	10–17
12,13-DiHOME	14.80	0.975	0.3	1.0	<4	3–6
9,10-DiHOME	15.18	0.981	0.5	1.8	<8	5–9
13-HODE	18.12	0.940	4.2	13.9	<9	7–16
9-HODE	18.25	0.942	0.2	0.5	<7	7–22
13-HpODE	18.60	0.939	3.5	11.8	<6	23–72
13-KODE	18.70	0.978	0.7	2.4	<14	8–48
9-HpODE	18.71	0.953	8.7	29.1	<7	29–72
9-KODE	19.09	0.951	3.2	10.8	<11	10–30
12(13)-EpOME	20.08	0.964	2.5	8.4	<7	14–39
9(10)-EpOME	20.27	0.932	0.6	1.9	<11	10–38

Dihomo-gamma-linolenic acid						
PGF1α	9.94	0.996	0.8	2.5	<14	5–12
15(S)-HETrE	19.41	0.988	0.5	1.7	<11	7–21

Alpha-linolenic acid						
9-HOTrE	16.57	0.967	0.3	1.1	<7	5–23

Eicosapentaenoic acid						
17,18-DiHETE	14.01	0.990	4.7	15.5	<11	8–26
14,15-DiHETE	14.49	0.993	0.8	2.7	<13	11–22
15(S)-HEPE	17.28	0.983	0.9	3.1	<13	11–19
12(S)-HEPE	17.67	0.985	0.3	0.9	<12	6–23
5(S)-HEPE	18.05	0.983	0.1	0.3	<12	8–24

Docosahexaenoic acid						
19,20-DiHDPA	15.60	0.991	1.7	5.7	<11	6–30
19(20)-EpDPE	19.93	0.988	1.2	4.0	<12	6–18

The table gives an overview about the acquired validation parameter linearity (*R* ^2^), sensitivity (LOD/LOQ) and reproducibility (precision and batch-to-batch effect). Validation data were achieved in pooled EDTA-plasma

#### Linearity and sensitivity

The matrix samples (pooled plasma) were spiked with academic standards (eight concentrations). We determined the linearity, LOD, and LOQ of each compound (104 targets). For the majority of the analytes, the *R* ^2^ ranged from 0.991 to 0.999, followed by 0.981–0.99 for 26 % and >0.9 for 17 % (Electronic Supplementary Material Table S-3). The range of LOQ was between 0.3 and 102 nM. For the majority of the targets (81 %), the LOQ was below 10 nM, for 56 % it was below 3 nM. Relatively high LOQs were observed for 5,6-Lipoxin A4 with values higher than 80 nM.

#### Reproducibility

To examine reproducibility, intra- and inter-batch variability was assessed for all academic standards. A total of three batches were processed. In each batch duplicate extractions and triplicate injections were performed. In Table 1, oxylipins detected in human plasma are shown. The reproducibility values for all compounds are summarized in the Electronic Supplementary Material (Table S-3). For intra-batch effects (precision), the highest RSD value above the LOD was taken. For the oxylipins, the RSD values ranged from 4 % up to 15 % with exceptions for 20-HETE (41 %), 9,12,13-TriHOME (32 %), and 16(17)-EpDPE (21 %). The batch-to-batch effects were higher for the low concentration level, compared to the high concentration level for the oxylipin platform. As problematic compounds with batch-to-batch effects higher than 50 %, 15-keto PGF_1α_, PGK_2_, Hepoxilin A_3_, 5,6-Lipoxin A_4_, LTE_4_, 5-HpETE, 12-HpETE, 15-HpETE, 9-HpODE, 13-HpODE, and PGF_3α_ need to be noted (Electronic Supplementary Material Table S-3).

#### Matrix effect

Matrix effect was evaluated by comparing extracts of spiked matrix with extracts of spiked blank matrix using water as blank matrix sample. Due to irreversible adsorption during SPE of the standards from water as compared to plasma, extreme low values (approximately 10 %) of the expected values were observed, prohibiting this approach to calculate matrix effects. Since water is not suitable as blank matrix, matrix variation tests are recommended before application to the various matrices. Although no matrix effect was determined, final concentrations were not effected as the calibration lines were made in matrix as well.

#### Recovery

**Fig. 4:**
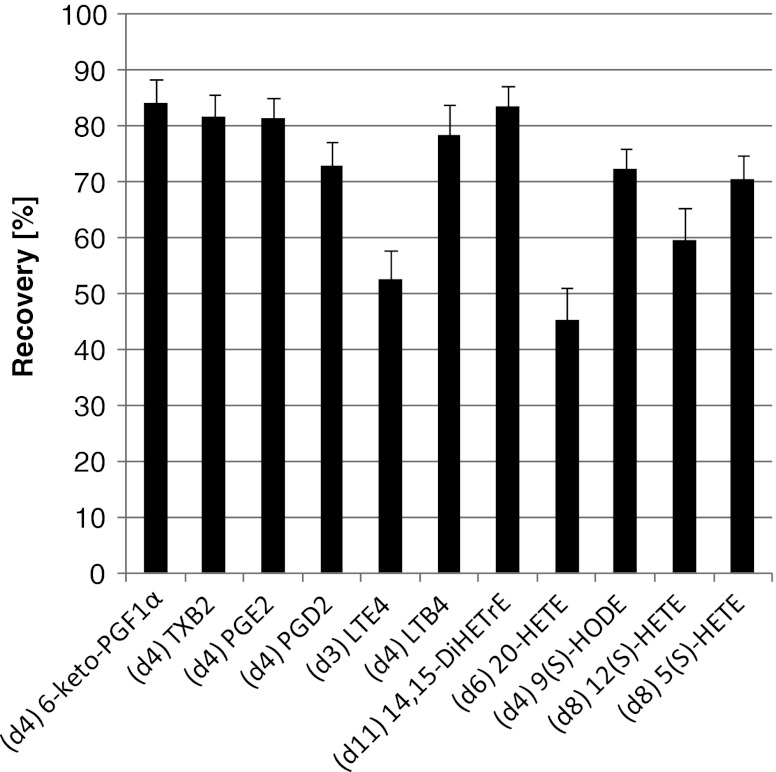
Recovery of deuterated, non-endogenous oxylipins; the recovery is shown as percentage value; the compounds are arranged by their polarity, with the more polar ones on the left hand side

For the recovery calculations, a set of deuterated ISTDs was used to prevent endogenous influences. Therefore, the standards were spiked either before or after the extraction and ratios of the peak areas were calculated. Recoveries ranged from 84 % (±4.4) to 45 % (±5.8), depending on the compound (Fig. 4). The recoveries for monohydroxy compounds (i.e., HETEs and HODEs) were lower than those of more polar prostaglandins and thromboxane. For (d3) LTE_4_, the recovery was lower than 60 %. In contrast to the other selected oxylipins, LTE_4_ contains a cysteinyl group, and like LTDs and LTCs, it belongs to the cysteinyl leukotriens. While recoveries differed between chemical class, inter-batch reproducibility was acceptable with RSD values between 4.3 % and 9.6 %, except for (d3) LTE4 and (d6) 20-HETE with more than 10 % (10.3 % and 12.9 %).

In summary, the analytical oxylipin platform developed here is characterized by high sensitivity reaching nanomolar levels, linearity with acceptable *R* ^2^, good reproducibility, and high coverage of oxylipin compounds.

### Detection of oxylipins in human plasma

Applying the oxylipin profiling platform to human EDTA-plasma, we were able to detect 36 oxylipins derived from 6 different PUFAs (Table 1). The majority of oxylipins detected in plasma are assigned to AA and LA, followed by metabolites derived from EPA, DHA, DGLA, and ALA. All these metabolites are biosynthesized via the three enzymatic COX, LOX, and CYP450 pathways. As the most prominent metabolites of the COX pathway, prostaglandins PGE_2_ and PGF_2α_ as well as the thromboxane B_2_, derived from AA, were detected. Furthermore, the prostaglandin F_1α_ was detected, which is generated from DGLA as precursor, while no EPA derived 3 series prostanes were detected. The hydroxylated products of AA, LA, ALA, and DGLA represent a large target group in this analytical profile and are regulated by diverse sources in biological systems. For example, hydroxy lipids are generated in the LOX pathways (see Fig. 1), the COX pathways (e.g., LA metabolism by COX to HODEs [47, 48], as well as through non-enzymatic interactions of the PUFAs with reactive oxygen [49]. In addition, both epoxide and diol products from the CYP epoxygenase-dependent metabolism of AA (e.g., 11,12-DiHETrE), LA (e.g., 9(10)-EpOME), EPA (e.g., 14,15-DiHETE), and DHA (19(20)-EpDPE and 19,20-DiHPDA) were detected (Table 2).

**Table 2:** Oxylipins detected in human plasma

LA	DGLA	AA	ALA	EPA	DHA
Prostanoids/thromboids					
	PGF1a	TXB2			
		PGF2a			
		PGE2			
		11b-PGE2			
		13,14-dihydro-PGF2a			

Diols					
12,13-DiHOME		14,15-DiHETrE		17,18-DiHETE	19,20-DiHDPA
9,10-DiHOME		11,12-DiHETrE		14,15-DiHETE	
		8,9-DiHETrE			
		5,6-DiHETrE			
		5,15-DiHETE^a^			
		8,15-DiHETE^a^			

Epoxides					
12(13)-EpOME		14(15)-EpETrE^a^			19(20)-EpDPE
9(10)-EpOME		11(12)-EpETrE^a^			16(17)-EpDPE^a^
		8(9)-EpETrE^a^			
		5(6)-EpETrE^a^			

Alcohols					
		12-HETE		12-HEPE	
9-HODE		5-HETE	9-HOTE	5-HEPE	
13-HODE	15-HETrE	15-HETE		15-HEPE	17-HDoHE^a^
		8-HETE			
		9-HETE^a^			
		20-HETE^a^			
		11-HETE			
		12-HHTrE^a^			

Ketones					
13-KODE		15-KETE^a^			
9-KODE		5-KETE^a^			

Hydroperoxides					
13-HpODE		15-HpETE^a^			
9-HpODE		5-HpETE^a^			

Triols					
9,12,13-TriHOME					
9,10,13-TriHOME					

Compounds are assigned to their specific precursor fatty acid

^a^Oxylipin metabolite was detected in pooled EDTA-plasma, but not in EDTA-plasma of the study samples

In the previous studies, it was demonstrated that a variety of oxylipins could be detected in human plasma [35, 36, 50, 51]. Our findings are in agreement with these reported profiles. In our hands though, remarkable variations were observed in profiles and actual concentration levels between plasma pools of different origin, sample handling, and storage (data not shown).

**Fig. 5:**
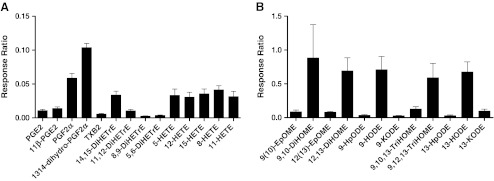
Baseline level of oxylipins detected in patients undergoing cardiac surgery (*n* = 5); the majority of assigned oxylipins are from two pathways, (**A**) metabolites derived from arachidonic acid (AA) and (**B**) metabolites generated from linoleic acid (LA); each graph shows the relative abundance of oxylipins, plotted as relative response ratios (obtained abundance corrected by appropriated ISTDs)

Comparing patient oxylipin concentrations before surgery revealed substantial variation between patients. Previous studies have shown that even when individuals have the same physical condition, gender, and health, their basal oxylipin concentrations can differ remarkably [52]. Nevertheless, we observed similar patterns in abundance and relationships between certain compounds (Fig. 5). For instance, the levels of PGF_2α_ and its metabolite 13,14-dihydro-PGF_2α_ were consistently higher in their relative abundance than levels of PGE_2_ and its epimer 11ß-PGE_2_. The levels of TXB_2_, the stable hydrolysis product of TXA_2_, were lower compared to the prostaglandins and thus indicate lower levels of TXA_2_. For CYP450 related compounds of the AA-pathway, 14,15-DiHETrE and 11,12-DiHETrE were found to be present at higher levels than 8,9-DiHETrE and 5,6-DiHETrE. Epoxides of the AA-pathway were below the detection limit and thus not detected in these samples. Also for the LA-pathway related oxylipins, a similarity in the basic abundance of the metabolites was observed. Although the epoxides and furthermore hydroperoxides originating from LA were detected above detection levels, they were less abundant compared to their subsequent derivatives.

### Alterations in oxylipin profiles during cardiac surgery

**Fig. 6:**
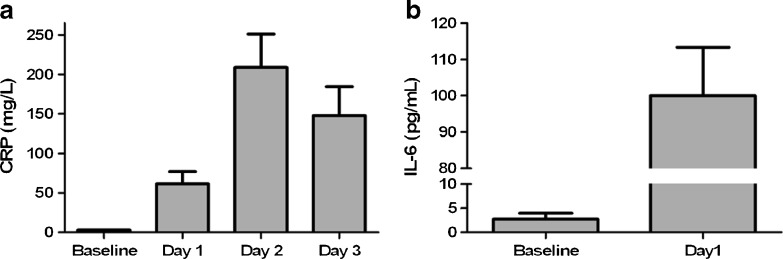
CRP- and IL-6 levels before and after surgery; (**a**) CRP levels (*n* = 5), after surgery the CRP levels increased dramatically; (**b**) IL-6 levels (*n* = 5), also for IL-6 levels a significant increment after surgery was observed

**Fig. 7:**
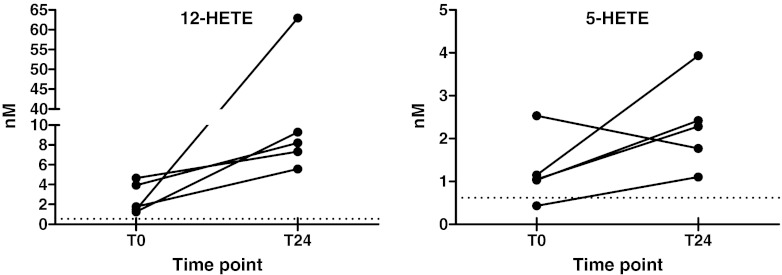
The pairplot demonstrates the abundance of the two monohydroxy fatty acids 12-HETE and 5-HETE at baseline levels before the surgery (T0) and 24 h after the surgery (T24) of the patients (*n* = 5); both oxylipins are enzymatically generated via lipoxygenase (12-LOX and 5-LOX, respectively) from arachidonic acid as precursor PUFA; *dotted lines* represent the limit of quantification

All included patients were gender and age matched, and underwent the same surgical procedure. Twenty-four hours after surgery, the levels of CRP and IL-6 were significantly increased indicating an inflammatory response after the surgery (Fig. 6). The metabolic state of the AA pathway before and after cardiac surgery was monitored for each patient (Electronic Supplementary Material Fig. 2). Despite substantial inter-patient variation in oxylipin concentrations both before and after surgery, several consistent changes in the oxylipin profiles were observed. For the HETEs, compounds biosynthesized via the LOX pathway, a dramatic increase was visible, in particular for 12-HETE and 5-HETE (Fig. 7). The LOX products 12-HETE and 5-HETE are involved in chemotaxis of neutrophils [9, 53–55]. Activation and accumulation of neutrophils play key roles in inflammatory processes during ischemia–reperfusion injury [53, 56, 57]. Due to its chemotactic properties, 12-HETE has been linked to healing processes during inflammation [58, 59]. Analysis of oxylipin profiles after abdominal aortic aneurism repair also showed high levels of 12-HETE in plasma 24 h after surgery for the group of patients which were in the resolution phase of inflammation [60].

**Fig. 8:**
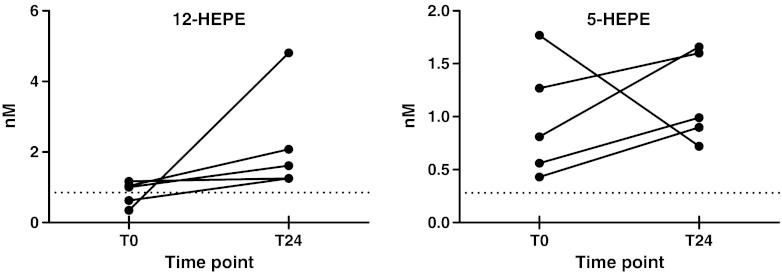
The pairplot demonstrates the abundance of the two monohydroxy fatty acids 12-HEPE and 5-HEPE at baseline levels before the surgery (T0) and 24 h after the surgery (T24) of the patients (*n* = 5); both are generated from eicosapentaenoic acid, biosynthesized by 12-LOX and 5-LOX; dotted lines represent the lower limit of quantification

Another 5-LOX generated compound LTB_4_ also promotes the chemotaxis of neutrophils [4, 6, 53, 56, 57, 61–64]. Interestingly, no LTB_4_ was detected in plasma while 5-HETE was one of the metabolites increased after surgery. Besides the high levels of 12-HETE and 5-HETE, increases in the analogous EPA metabolites 12-HEPE and 5-HEPE were observed (Fig. 8). The metabolites 12-HEPE and 5-HEPE are produced via the LOX pathway, but with EPA as precursor fatty acid. EPA derived LOX products can provide chemotactic activities, but are less potent than products of AA [62, 65].

For COX derived metabolites, such as the prostaglandins and thromboxanes, no significant changes were observed in human plasma (Electronic Supplementary Material Fig. 2). In previous studies on ischemia/reperfusion of the heart, metabolites of the COX pathway, especially PGE_2_, TXB_2_, and 6-keto-PGF_1α_, were significantly increased in heart perfusates [37]. A similar observation was made for CYP450 related compounds such as DiHETrEs. At the 24 h time point sampled here, only minor fluctuations were observed, suggesting acute COX-dependent responses had subsided by this time.

Our findings indicate that the analytical platform developed here allows the detection of consistent alterations in oxylipin profiles, even within a small patient population (*n* = 5).

## Conclusions

We developed an LC-MS/MS method to detect oxylipins. Our target library comprises an expanded set of more than 100 compounds biosynthesized from six different PUFAs in one LC-MS/MS analysis. These targets include a wide array of both pro- and anti-inflammatory lipid mediators. Our oxylipin profiling platform was developed and validated for human plasma on a triple quadrupole mass spectrometer using multiple reaction monitoring operated in a dynamic MRM mode to guarantee high sensitivity, i.e., down to nM levels. This technology allows the sensitive detection of co-eluting compounds and isomers as well as several isobaric compounds by detecting specific transitions for each analyte with optimal dwell times in defined time windows. Our method was demonstrated on patients undergoing cardiac surgery and was able to detect one third of our target library in human plasma. The metabolites 12-HETE and 5-HETE, known to be related to cardiac surgery and healing processes, were consistently increased after cardiac surgery. In addition, an increase of similar monohydroxy metabolites derived from EPA was observed. In conclusion, the analytical platform developed here allows specific and sensitive quantitative assessment of more than 100 oxylipins and may have wider applications in studies on the role of these highly bioactive metabolites in human diseases. The successful application of the developed method to this biological study implies that the developed platform can be used broadly to profile these highly bioactive compounds.

## Electronic Supplementary Materials

Below is the link to the electronic supplementary material.

ESM 1 (PDF 878 kb)
