# Supplementary material for: Quantitative profiling of oxylipins through comprehensive LC-MS/MS analysis: application in cardiac surgery
Source: Anal Bioanal Chem. 2012 Jul 20;404(5):1413–26. doi: 10.1007/s00216-012-6226-x (PMC3426673; doi:10.1007/s00216-012-6226-x)
Supplement: Supplementary file 1 — (PDF 878 kb) [file 216_2012_6226_MOESM1_ESM.pdf]

Analytical and Bioanalytical Chemistry
Electronic Supplementary Material

Quantitative profiling of oxylipins through comprehensive LC-MS/MS analysis: application in cardiac surgery

Katrin Strassburg, Annemarie M.L. Huijbrechts, Kirsten A. Kortekaas, Jan H. Lindeman, Theresa L. Pedersen, Adrie Dane, Ruud Berger, Arjan Brenkman, Thomas Hankemeier, John van Duynhoven, Eric Kalkhoven, John W. Newman and Rob J.Vreeken

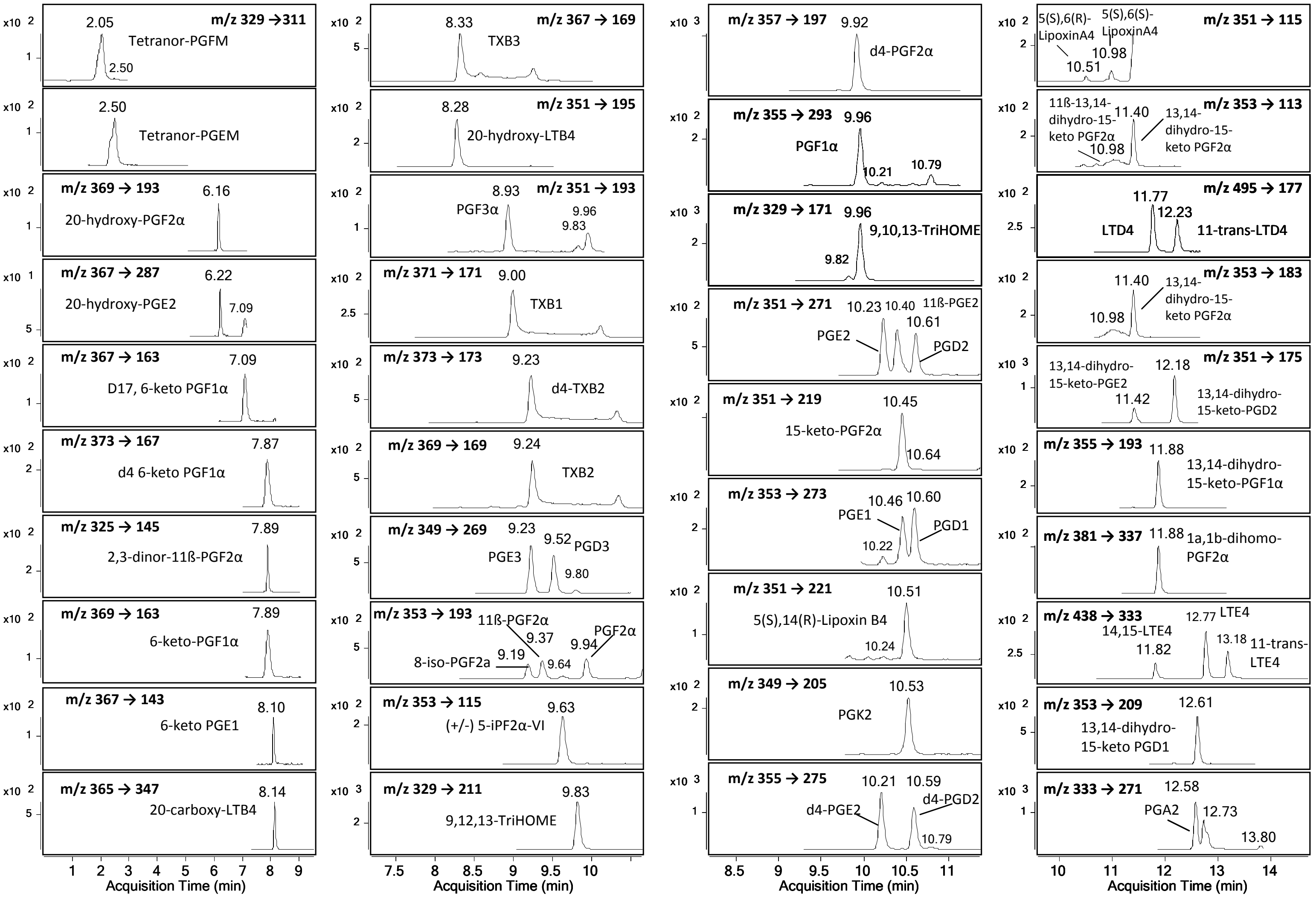

Fig. S-1a LC-MS/MS extracted chromatograms of oxylipin library in the range from 2 to 13. 8 min

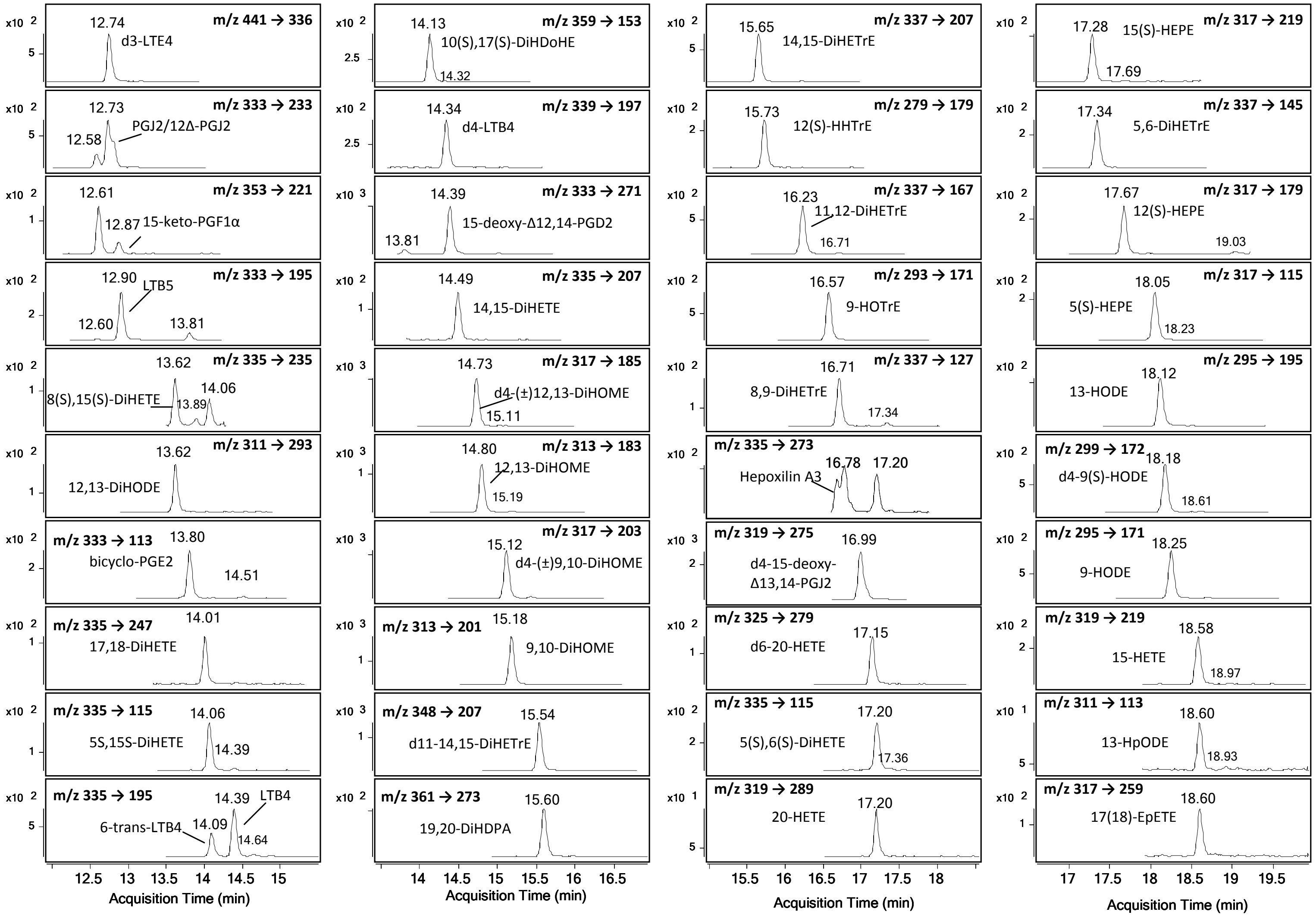

Fig. S-1b LC-MS/MS extracted chromatograms of oxylipin library in the range from 10.51 to 18.6 min

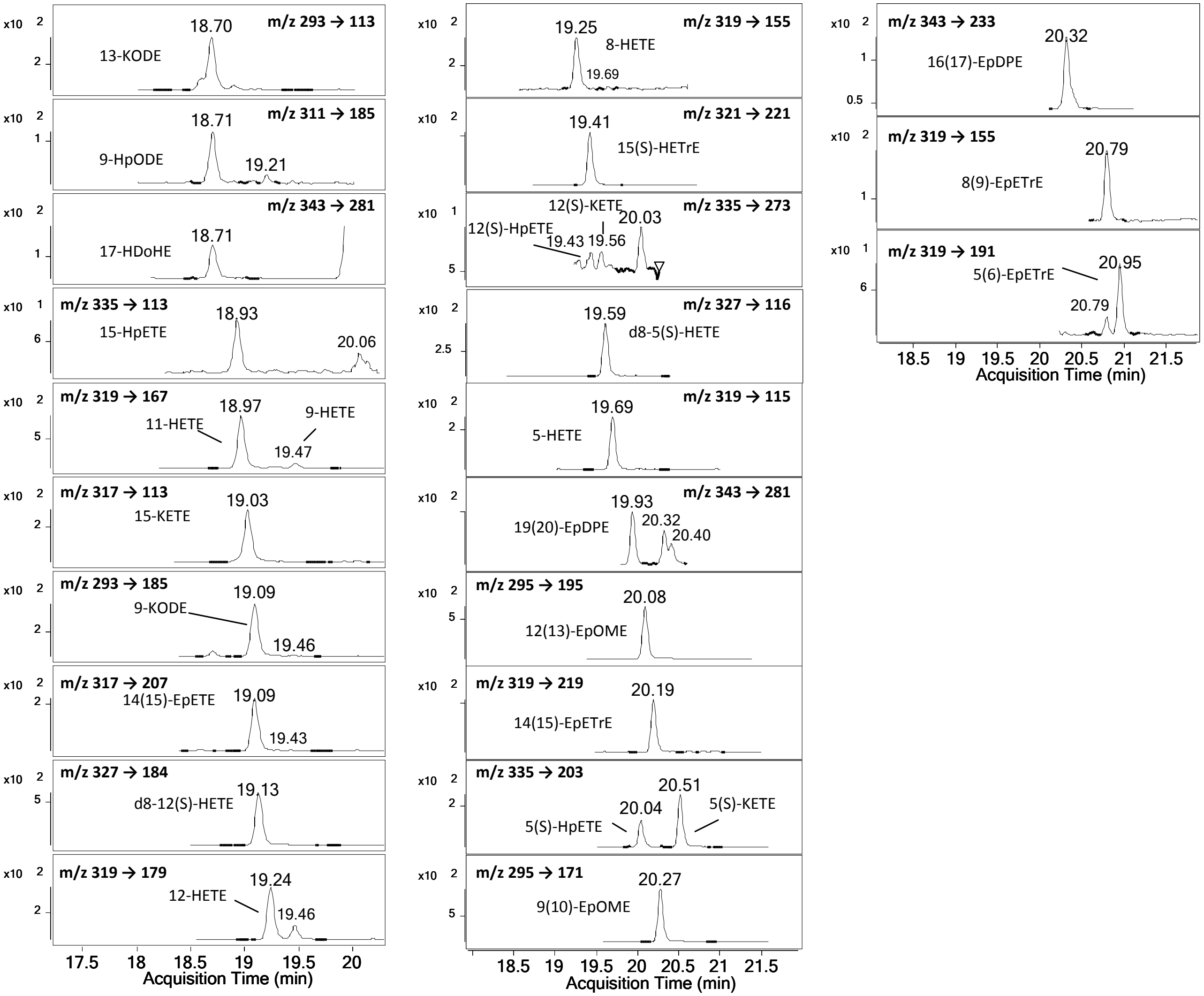

Fig. S-1c LC-MS/MS extracted chromatograms of oxylipin library in the range from 15.65 to 20.95 min

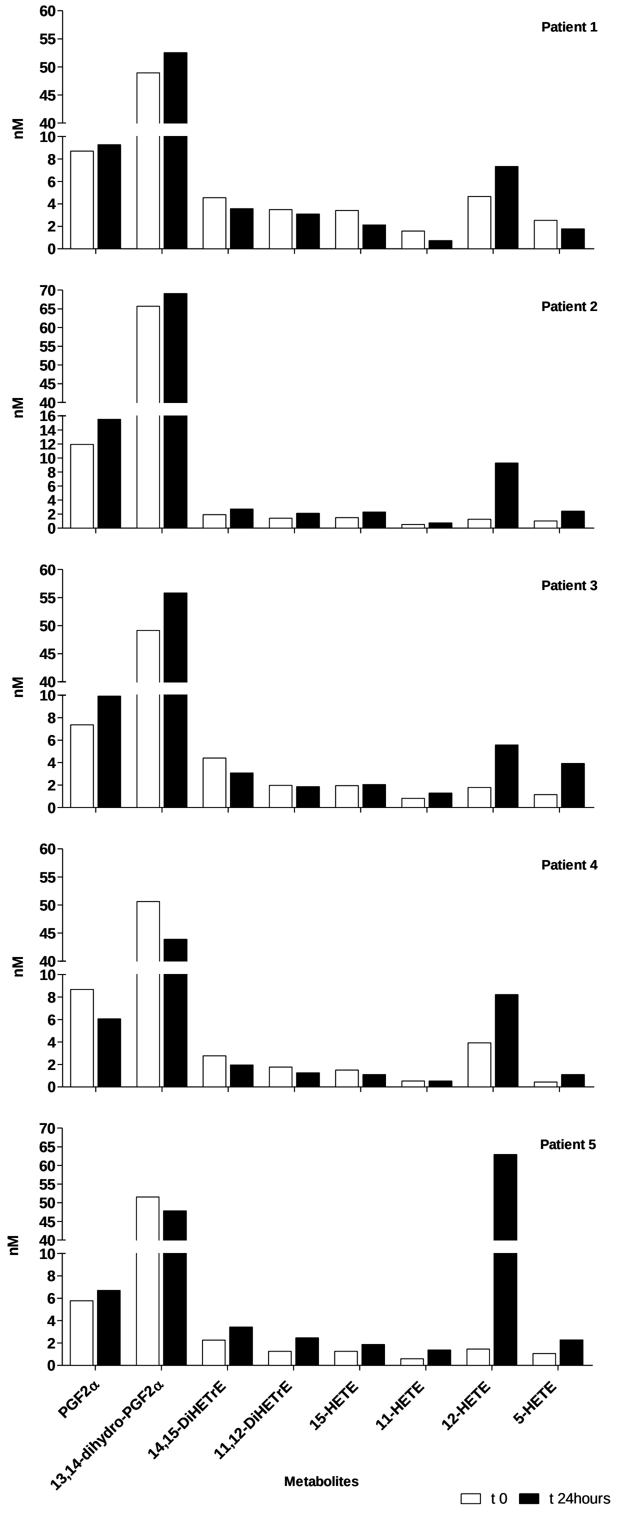

Fig. S-2 Oxylipins generated in the arachidonic acid pathway with their abundance in the patients (n=5, presented as Patient 1 to Patient 5); the baseline level before surgery (white bars) and levels 24h after the surgery (black bars) are shown

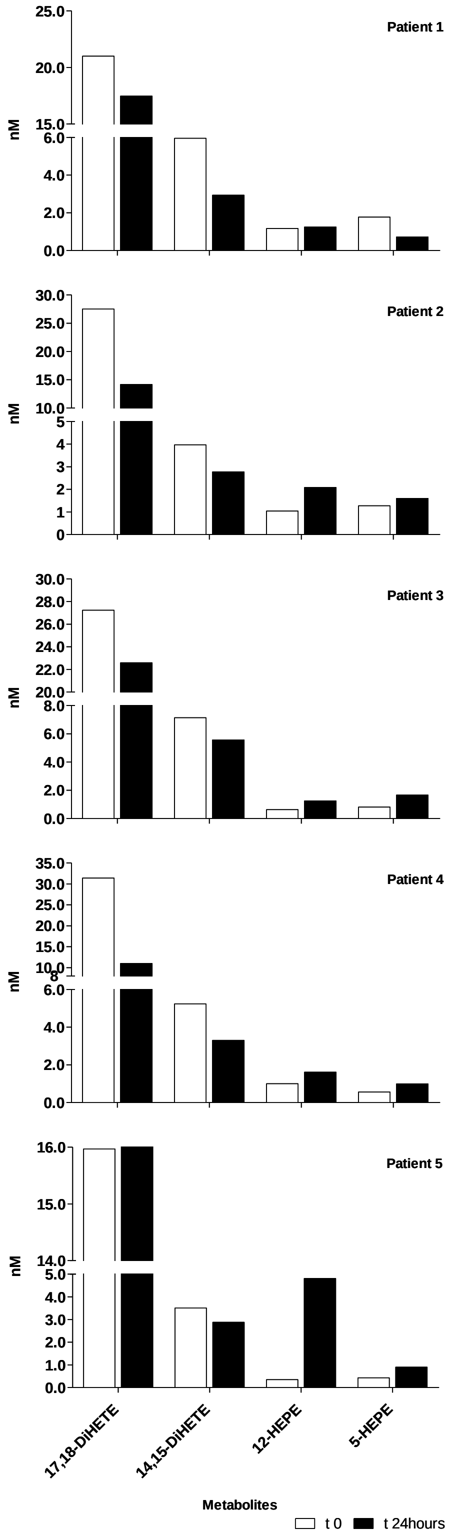

Fig. S-3 Oxylipins generated in the eicosapentaenoic acid pathway with their abundance in the patients (n=5, presented as Patient 1 to Patient 5); the baseline level before surgery (white bars) and levels 24h after the surgery (black bars) are shown

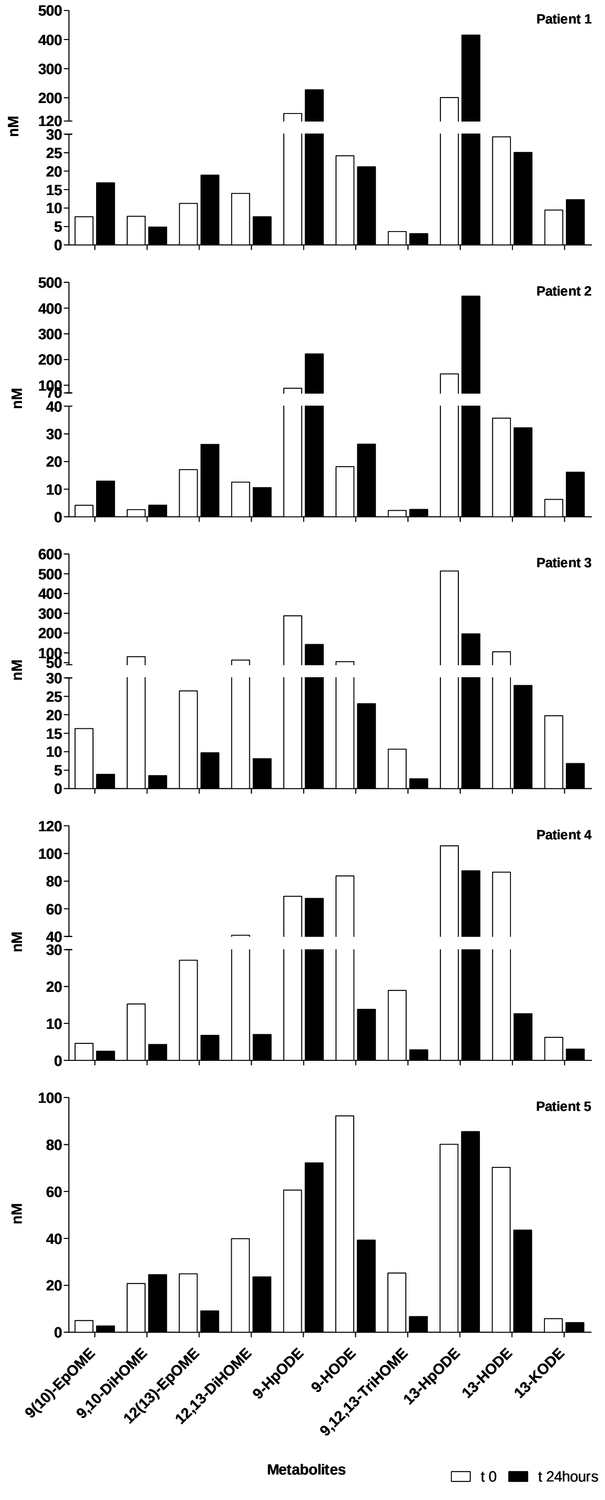

Fig. S-4 Oxylipins generated in the linoleic acid pathway with their abundance in the patients (n=5, presented as Patient 1 to Patient 5); the baseline level before surgery (white bars) and levels 24h after the surgery (black bars) are shown

Table S-1 Oxylipin library. The table contains 104 compounds considered in the validation with information of precursor and product ions, as well as retention time and the ISTD for correction.

Compound Name	Lipid Maps ID	Precursor Ion	Product Ion	Retention Time	Internal Standard
Tetranor-PGFM	LMFA03010139	329.2	311.2	2.05	(d4) PGF2α
Tetranor-PGEM	LMFA03010032	327.1	309.2	2.50	(d4) PGE2
20-hydroxy PGF2α	LMFA03010029	369.2	193.1	6.16	(d4) PGF2α
20-hydroxy PGE2	LMFA03010014	367.2	287.2	6.22	(d4) PGE2
Δ17-6-keto PGF1α	LMFA03010149	367.2	163.1	7.09	(d4) 6-keto PGF1α
**(d4) 6-keto PGF1α**	LMFA03010037	373.2	167.2	7.87	-
2,3-dinor-11b PGF2α	LMFA03010011	325.2	145.1	7.89	(d4) PGF2α
6-keto PGF1α	LMFA03010001	369.2	163.1	7.89	(d4) 6-keto PGF1α
6-keto PGE1	LMFA03010012	367.2	143.1	8.10	(d4) PGE2
20-carboxy LTB4	LMFA03020016	365.2	347.2	8.14	(d4) LTB4
20-hydroxy LTB4	LMFA03020018	351.2	195.1	8.28	(d4) LTB4
TXB3	LMFA03030006	367.2	169.1	8.33	(d4) TXB2
PGF3α	LMFA03010138	351.2	193.2	8.93	(d4) PGF2α
TXB1	LMFA03030008	371.2	171.1	9.04	(d4) TXB2
**(d4) TXB2**	LMFA03030010	373.2	173.1	9.23	-
8-iso PGF2α	LMFA03110001	353.2	193.2	9.23	(d4) PGF2α
PGE3	LMFA03010135	349.2	269.2	9.24	(d4) PGE2
TXB2	LMFA03030002	369.2	169.1	9.24	(d4) TXB2
11β-PGF2α	LMFA03010036	353.2	193.2	9.37	(d4) PGF2α
PGD3	LMFA03010142	349.2	269.2	9.52	(d4) PGD2
(+/-) 5-iPF2α-VI	LMFA03110011	353.2	115.1	9.63	(d4) PGF2α
9,12,13-TriHOME	LMFA02000014	329.2	211.2	9.83	(d4) 9(S)-HODE
**(d4) PGF2α**	LMFA03010006	357.3	197.2	9.92	-
PGF2α	LMFA03010002	353.2	193.2	9.94	(d4) PGF2α
9,10,13-TriHOME	-	329.2	171.1	9.96	(d4) 9(S)-HODE
PGF1α	LMFA03010137	355.2	293.2	9.96	(d4) PGF2α
**(d4) PGE2**	LMFA03010007	355.2	275.2	10.21	-
PGE2	LMFA03010003	351.2	271.2	10.23	(d4) PGE2
11β-PGE2	LMFA03010060	351.2	271.2	10.40	(d4) PGE2
15-keto PGF2α	LMFA03010026	351.2	219.1	10.45	(d4) PGF2α
PGE1	LMFA03010134	353.2	273.2	10.46	(d4) PGE2
5(S),14(R)-Lipoxin B4	LMFA03040002	351.2	221.2	10.51	(d4) LTB4
5(S),6(R)-Lipoxin A4	LMFA03040001	351.2	115.1	10.51	(d4) LTB4
PGK2	LMFA03010023	349.2	205.1	10.53	(d4) PGE2
**(d4) PGD2**	LMFA03010008	355.2	275.2	10.59	-
PGD1	LMFA03010049	353.2	273.2	10.60	(d4) PGD2
PGD2	LMFA03010004	351.2	271.2	10.61	(d4) PGD2
15-keto PGF1α	LMFA03010150	353.2	221.1	10.70	(d4) 6-keto PGF1α
13,14-dihydro PGF2α	LMFA03010079	355.2	275.2	10.79	(d4) PGF2α
11β-13,14-dihydro-15-keto PGF2α	LMFA03010203	353.2	113.2	10.98	(d4) PGF2α
5(S),6(S)-Lipoxin A4	LMFA03040003	351.2	115.1	10.98	(d4) LTB4
13,14-dihydro-15-keto PGF2α	LMFA03010027	353.2	183.1	11.40	(d4) PGF2α
13,14-dihydro-15-keto PGE2	LMFA03010031	351.2	175.2	11.42	(d4) PGE2
14,15-LTC4	LMFA03020031	624.3	272.1	11.48	(d3) LTE4

Continue Table S-1

Compound Name	Lipid Maps ID	Precursor Ion	Product Ion	Retention Time	Internal Standard
LTD4	LMFA03020006	495.2	177.1	11.77	(d3) LTE4
14,15-LTE4	LMFA03020033	438.2	333.2	11.82	(d3) LTE4
13,14-dihydro-15-keto PGF1α	-	355.2	193.2	11.88	(d4) PGF2α
1α,1b-dihomo PGF2α	LMFA03010157	381.3	337.2	11.88	(d4) PGF2α
13,14-dihydro-15-keto PGD2	LMFA03010022	351.2	175.2	12.18	(d4) PGD2
11-trans LTD4	LMFA03020021	495.2	177.1	12.23	(d3) LTE4
PGA2	LMFA03010035	333.2	271.2	12.58	(d4) PGE2
13,14-dihydro-15-keto PGD1	-	353.2	209.1	12.61	(d4) PGD2
PGJ2	LMFA03010019	333.2	233.1	12.73	(d4) PGD2
Δ12-PGJ2	LMFA03010020	333.2	233.1	12.73	(d4) 15-deoxy-Δ12,14-PGJ2
**(d3) LTE4**	-	441.2	336.2	12.74	-
LTE4	LMFA03020002	438.2	333.2	12.77	(d3) LTE4
LTB5	LMFA03020010	333.2	195.1	12.90	(d4) LTB4
LTC4	LMFA03020003	624.3	272.1	13.04	(d3) LTE4
11-trans LTE4	LMFA03020022	438.2	333.2	13.18	(d3) LTE4
11-trans LTC4	LMFA03020020	624.3	272.1	13.57	(d3) LTE4
12,13-DiHODE	LMFA02000046	311.2	293	13.62	(d4) 9(S)-HODE
8(S),15(S)-DiHETE	LMFA03060050	335.2	235.2	13.62	(d4) LTB4
bicyclo-PGE2	LMFA03010034	333.2	113.2	13.80	(d4) PGE2
17,18-DiHETE	LMFA03060078	335.2	247.2	14.01	(d11) 14,15-DiHETrE
5(S),15(S)-DiHETE	LMFA03060049	335.2	115.2	14.06	(d4) LTB4
6-trans-LTB4	LMFA03020013	335.2	195.1	14.09	(d4) LTB4
10(S),17(S)-DiHDoHE	LMFA04000047	359.2	153.2	14.13	(d8) 12(S)-HETE
**(d4) LTB4**	LMFA03020030	339.2	197.1	14.34	-
15-deoxy-Δ12,14-PGD2	LMFA03010051	333.2	271.2	14.39	(d4) PGD2
LTB4	LMFA03020001	335.2	195.1	14.39	(d4) LTB4
14,15-DiHETE	LMFA03060077	335.2	207.1	14.49	(d11) 14,15-DiHETrE
**(d4)(±)12,13-DiHOME**	LMFA01050357	317.3	185.2	14.73	-
12,13-DiHOME	LMFA01050351	313.2	183.2	14.80	(d4) 12,13-DiHOME
**(d4)-(±)9,10-DiHOME**	LMFA01050358	317.3	203.2	15.12	-
9,10-DiHOME	LMFA01050350	313.2	201.1	15.18	(d4) 9,10-DiHOME
**(d11) 14,15-DiHETrE**	-	348.3	207.1	15.54	-
19,20-DiHDPA	LMFA04000043	361.2	273.3	15.60	(d8) 12(S)-HETE
14,15-DiHETrE	LMFA03050010	337.2	207.2	15.65	(d11) 14,15-DiHETrE
12S-HHTrE	LMFA03050002	279.2	179.2	15.73	(d8) 12(S)-HETE
11,12-DiHETrE	LMFA03050008	337.2	167.2	16.23	(d11) 14,15-DiHETrE
9-HOTrE	LMFA02000024	293.2	171.1	16.57	(d4) 9(S)-HODE
8,9-DiHETrE	LMFA03050006	337.2	127	16.71	(d11) 14,15-DiHETrE
Hepoxilin A3	LMFA03090005	335.2	273.2	16.78	(d8) 12(S)-HETE
**(d4) 15-deoxy-Δ12,14-PGJ2**	LMFA03010177	319.2	275.3	16.99	-
**(d6) 20-HETE**	LMFA03060082	325.3	279.2	17.15	-
20-HETE	LMFA03060009	319.2	289.2	17.20	d6-20-HETE
5(S),6(S)-DiHETE	LMFA03060018	335.2	115.1	17.20	(d4) LTB4

Continue Table S-1

Compound Name	Lipid Maps ID	Precursor Ion	Product Ion	Retention Time	Internal Standard
15(S)-HEPE	LMFA03070009	317.2	219.2	17.28	(d8) 5(S)-HETE
5,6-DiHETrE	LMFA03050004	337.2	145.1	17.34	(d11) 14,15-DiHETrE
12(S)-HEPE	LMFA03070008	317.2	179.1	17.67	(d8) 12(S)-HETE
5(S)-HEPE	LMFA03070010	317.2	115.1	18.05	(d8) 5(S)-HETE
13-HODE	LMFA01050349	295.2	195.2	18.12	(d4) 9(S)-HODE
**(d4) 9(S)-HODE**	LMFA01050353	299.2	172.1	18.18	-
9-HODE	LMFA01050278	295.2	171.1	18.25	(d4) 9(S)-HODE
15-HETE	LMFA03060001	319.2	219.2	18.58	(d8) 5(S)-HETE
13-HpODE	LMFA02000034	311.2	113.2	18.60	(d4) 9(S)-HODE
17(18)-EpETE	LMFA03000004	317.2	259.2	18.60	(d11) 14,15-DiHETrE
13-KODE	LMFA02000016	293.2	113.1	18.70	(d4) 9(S)-HODE
9-HpODE	LMFA02000012	311.2	185.2	18.71	(d4) 9(S)-HODE
17-HDoHE	LMFA04000032	343.2	281.3	18.71	(d8) 12(S)-HETE
15-HpETE	LMFA03060014	335.2	113.1	18.93	(d8) 5(S)-HETE
11-HETE	LMFA03060028	319.2	167.1	18.97	(d8) 12(S)-HETE
15-KETE	LMFA03060051	317.2	113.2	19.03	(d8) 5(S)-HETE
9-KODE	LMFA01060177	293.2	185.2	19.09	(d4) 9(S)-HODE
14(15)-EpETE	LMFA03000003	317.2	207.1	19.09	(d11) 14,15-DiHETrE
**(d8) 12(S)-HETE**	LMFA03060081	327.3	184.2	19.13	-
12-HETE	LMFA03060088	319.2	179.2	19.24	(d8) 12(S)-HETE
8-HETE	LMFA03060006	319.2	155.1	19.25	(d8) 5(S)-HETE
15(S)-HETrE	LMFA03050007	321.2	221.2	19.41	(d11) 14,15-DiHETrE
12(S)-HpETE	LMFA03060013	335.2	273.3	19.43	(d8) 12(S)-HETE
9-HETE	LMFA03060089	319.2	167.1	19.47	(d8) 12(S)-HETE
12-KETE	LMFA03060019	317.2	273.3	19.56	(d8) 12(S)-HETE
**(d8) 5(S)-HETE**	LMFA03060005	327.3	116.1	19.59	-
5-HETE	LMFA03060002	319.2	115.1	19.69	(d8) 5(S)-HETE
19(20)-EpDPE	LMFA04000038	343.2	281.3	19.93	(d8) 12(S)-HETE
5(S)-HpETE	LMFA03060012	335.2	203.2	20.04	(d8) 5(S)-HETE
12(13)-EpOME	LMFA02000038	295.2	195.2	20.08	(d4) 12,13-DiHOME
14(15)-EpETrE	LMFA03080005	319.2	219.2	20.19	(d11) 14,15-DiHETrE
9(10)-EpOME	LMFA02000037	295.2	171.2	20.27	(d4) 9,10-DiHOME
16(17)-EpDPE	LMFA04000037	343.2	233.2	20.32	(d8) 12(S)-HETE
5-KETE	LMFA03060011	317.2	203.2	20.51	(d8) 5(S)-HETE
11(12)-EpETrE	LMFA03080004	319.2	167.1	20.71	(d11) 14,15-DiHETrE
8(9)-EpETrE	LMFA03080003	319.2	155.1	20.79	(d11) 14,15-DiHETrE
5(6)-EpETrE	LMFA03080002	319.2	191.2	20.95	(d11) 14,15-DiHETrE

Table S-2 Concentration levels of 104 oxylipins for calibration lines. The values display the amount on column.

	conc [nM]								
**compound name**	**C0**	**C1**	**C2**	**C3**	**C4**	**C5**	**C6**	**C7**	**C8**
Tetranor-PGFM	0	3	6	13	25	50	101	201	403
Tetranor-PGEM	0	3	6	13	25	50	100	201	401
20-hydroxy PGF2α	0	2	4	9	18	35	71	142	284
20-hydroxy PGE2	0	3	6	12	25	50	100	200	399
Δ17-6-keto PGF1α	0	2	4	8	17	33	67	133	266
2,3-dinor-11b PGF2α	0	3	6	13	25	51	102	204	408
6-keto PGF1α	0	2	4	9	18	35	71	142	284
20-carboxy LTB4	0	2	4	9	17	34	69	138	275
6-keto PGE1	0	3	6	12	24	48	95	190	380
20-hydroxy LTB4	0	2	4	9	17	35	70	139	278
TXB3	0	3	6	12	25	50	100	200	399
PGF3α	0	2	4	9	17	35	70	139	278
TXB1	0	3	6	12	24	47	94	188	376
8-iso PGF2α	0	2	4	9	17	35	69	138	277
PGE3	0	3	6	12	25	50	100	200	400
TXB2	0	3	6	12	24	47	95	189	378
11β-PGF2α	0	3	6	12	25	49	99	198	395
PGD3	0	3	6	12	25	50	100	200	400
5-iPF2α-VI	0	2	4	9	18	35	70	140	281
9,12,13-TriHOME	0	3	6	13	25	50	100	201	401
PGF1α	0	2	4	9	17	34	69	138	275
9,10,13-TriHOME	0	3	6	13	25	50	101	201	402
PGF2α	0	2	4	9	17	35	69	138	277
PGE2	0	2	4	9	17	35	70	139	278
11β-PGE2	0	3	6	12	25	50	99	199	397
15-keto PGF2α	0	3	6	12	25	50	99	199	397
PGE1	0	2	4	9	17	35	69	138	277
PGK2	0	2	4	9	17	35	70	140	280
5(S),14(R)-Lipoxin B4	0	2	4	9	17	35	70	139	278
PGD1	0	3	6	12	25	49	99	198	395
PGD2	0	2	4	9	17	35	70	139	278
15-keto PGF1α	0	2	4	9	17	35	69	138	277
13,14-dihydro PGF2α	0	3	6	12	25	49	98	196	393
11β-13,14-dihydro-15-keto PGF2α	0	2	4	9	17	35	69	138	277
LTD4	0	2	4	8	17	34	68	135	271
13,14-dihydro-15-keto PGF2α	0	3	6	12	25	49	99	198	395
14,15-LTC4	0	3	6	13	25	50	101	202	403
5(S),6(R)-Lipoxin A4	0	5	9	18	36	73	146	291	583
13,14-dihydro-15-keto PGE2	0	3	6	12	25	50	99	199	397
5(S),6(S)-Lipoxin A4	0	2	4	9	17	35	70	139	278
14,15-LTE4	0	3	6	13	25	50	101	201	403
11-trans LTD4	0	2	4	8	17	34	68	135	271
13,14-dihydro-15-keto PGF1α	0	3	6	12	24	48	95	190	380
1a,1b-dihomo PGF2α	0	3	6	11	23	46	92	183	366
13,14-dihydro-15-keto PGD2	0	3	6	12	25	50	99	199	397
PGA2	0	3	7	13	26	52	105	209	419
13,14-dihydro-15-keto PGD1	0	2	4	9	17	35	69	138	276
PGJ2	0	3	7	13	26	52	105	209	419
LTE4	0	3	6	13	25	50	100	201	402
Δ12-PGJ2	0	2	3	7	13	26	52	105	209
LTB5	0	2	4	9	18	35	70	140	281
LTC4	0	3	6	13	25	50	101	202	403
11-trans LTE4	0	3	6	13	25	50	100	201	402
11-trans LTC4	0	3	6	13	25	50	101	202	403

Continue Table S-2

	conc [nM]								
**compound name**	**C0**	**C1**	**C2**	**C3**	**C4**	**C5**	**C6**	**C7**	**C8**
12,13-DiHODE	0	2	4	8	17	34	67	135	269
8(S),15(S)-DiHETE	0	2	4	9	17	35	70	139	279
bicyclo-PGE2	0	3	7	13	26	52	105	209	419
17,18-DiHETE	0	3	6	12	25	50	100	200	400
5(S),15(S)-DiHETE	0	2	4	9	17	34	69	137	275
6-trans-LTB4	0	2	4	9	17	34	69	137	275
10(S),17(S)-DiHDoHE	0	2	4	9	17	35	70	140	280
15-deoxy-Δ12,14-PGD2	0	3	5	10	20	41	81	163	326
LTB4	0	4	9	17	35	70	139	279	558
14,15-DiHETE	0	3	6	12	25	50	100	200	400
12,13-DiHOME	0	2	4	9	17	35	69	138	276
9,10-DiHOME	0	2	4	9	17	35	69	138	276
19,20-DiHDPA	0	2	4	9	17	35	70	139	278
14,15-DiHETrE	0	2	5	9	18	37	74	148	295
12S-HHTrE	0	2	4	9	17	35	70	140	280
11,12-DiHETrE	0	2	5	9	19	37	74	149	297
9-HOTrE	0	2	4	9	18	36	71	143	285
8,9-DiHETrE	0	2	4	9	17	35	70	140	280
Hepoxilin A3	0	2	4	9	17	35	70	139	279
20-HETE	0	2	4	8	17	33	66	132	265
5(S),6(S)-DiHETE	0	2	4	9	17	34	69	137	275
15(S)-HEPE	0	2	4	9	18	35	70	141	282
5,6-DiHETrE	0	2	5	9	19	37	74	149	297
12(S)-HEPE	0	2	4	9	18	35	70	141	282
5(S)-HEPE	0	2	4	9	18	35	70	141	282
13-HODE	0	2	4	9	18	35	71	142	284
9-HODE	0	2	4	9	18	35	71	142	284
15-HETE	0	2	4	8	17	33	67	133	266
13-HpODE	0	4	7	14	28	56	112	224	448
17(18)-EpETE	0	2	4	9	18	35	70	141	282
13-KODE	0	2	4	9	17	34	69	138	276
9-HpODE	0	2	4	8	17	34	67	135	269
17-HDoHE	0	2	4	9	18	36	71	142	285
15-HpETE	0	2	5	9	18	36	73	145	290
11-HETE	0	2	4	9	17	35	70	140	280
15-KETE	0	2	4	9	17	35	70	140	280
9-KODE	0	2	4	9	17	34	69	138	276
14(15)-EpETE	0	2	4	8	17	33	66	132	265
12-HETE	0	2	4	9	18	35	70	141	282
8-HETE	0	2	4	9	17	35	70	140	280
15(S)-HETrE	0	2	4	9	17	35	70	140	280
12S-HpETE	0	2	5	9	18	36	73	145	290
9-HETE	0	2	4	9	17	35	70	140	280
12-KETE	0	2	4	9	18	35	70	141	282
5-HETE	0	2	4	9	18	35	70	141	282
19(20)-EpDPE	0	2	4	9	18	36	71	142	285
5(S)-HpETE	0	2	5	9	18	36	72	144	289
12(13)-EpOME	0	2	4	9	17	34	69	137	274
14(15)-EpETrE	0	2	4	9	17	35	70	140	280
9(10)-EpOME	0	2	4	9	17	34	69	137	274
16(17)-EpDPE	0	3	6	13	25	51	102	203	407
5-KETE	0	1	2	4	8	16	32	64	127
11(12)-EpETrE	0	2	4	9	17	35	70	140	280
8(9)-EpETrE	0	3	6	13	25	50	101	201	402
5(6)-EpETrE	0	2	5	10	19	38	77	153	306

Table S-3 Statistics of the validation, including linearity (R^2), sensitivity (LOD/LOQ), reproducibility (precision and batch-to-batch effect) for the oxylipin library

Compound Name	Retention Time	R^2	LOD [nM]	LOQ [nM]	Precision RSD [%]	Batch-to-Batch Effect RSD [%]
Tetranor-PGFM	2.05	0.985	0.6	2.1	< 16	11-28
Tetranor-PGEM	2.50	0.995	1.2	4.0	< 10	5-17
20-hydroxy PGF2α	6.16	0.992	0.6	1.9	< 15	6-15
20-hydroxy PGE2	6.22	0.994	1.4	4.5	< 17	6-29
Δ17-6-keto PGF1α	7.09	0.993	0.5	1.5	< 7	35-38
2,3-dinor-11b PGF2α	7.89	0.998	0.2	0.7	< 11	4-12
6-keto PGF1α	7.89	0.994	0.8	2.6	< 10	3-16
20-carboxy LTB4	8.10	0.991	3.6	12.1	< 17	7-23
6-keto PGE1	8.14	0.993	0.5	1.6	< 6	3-22
20-hydroxy LTB4	8.28	0.997	0.7	2.4	< 9	6-26
TXB3	8.33	0.989	0.2	0.6	< 12	8-30
PGF3α	8.93	0.992	2.7	9.0	< 25	5-67
TXB1	9.04	0.989	0.4	1.3	< 13	6-33
8-iso PGF2α	9.23	0.995	1.0	3.2	< 17	4-18
PGE3	9.24	0.992	0.5	1.7	< 14	5-26
TXB2	9.24	0.995	0.3	1.0	< 7	13-22
11β-PGF2α	9.37	-	-	-	-	-
PGD3	9.52	0.993	0.5	1.6	< 16	6-16
5-iPF2a-VI	9.63	0.994	0.4	1.5	< 14	8-16
9,12,13-TriHOME	9.83	0.946	0.2	0.8	< 32	9-16
PGF1α	9.94	0.996	0.8	2.5	< 14	5-12
9,10,13-TriHOME	9.96	0.953	0.5	1.8	< 10	10-17
PGF2α	9.96	0.998	0.9	2.9	< 10	4-15
PGE2	10.23	0.999	0.2	0.6	< 13	4-20
11β-PGE2	10.40	0.998	0.9	3.1	< 8	3-19
15-keto PGF2α	10.45	0.998	0.5	1.7	< 12	4-12
PGE1	10.46	0.995	1.7	5.6	< 26	4-28
5(S),14(R)-Lipoxin B4	10.51	0.990	2.6	8.5	< 18	7-21
5(S),6(R)-Lipoxin A4	10.51	0.983	26.8	89.4	< 43	12-53
PGK2	10.53	0.373	21.6	72.1	< 87	45-70
PGD1	10.60	0.960	2.1	7.0	< 24	5-25
PGD2	10.61	0.994	0.3	0.9	< 11	6-18
15-keto PGF1α	10.70	0.995	1.0	3.3	< 15	3-11
13,14-dihydro PGF2α	10.79	0.990	11.4	38.2	< 8	12-26
11β-13,14-dihydro-15-keto PGF2α	10.98	0.997	5.7	18.9	< 44	9-28
5(S),6(S)-Lipoxin A4	10.98	0.987	30.6	102.0	< 23	8-21
13,14-dihydro-15-keto PGF2α	11.40	0.996	1.0	3.5	< 19	8-14
13,14-dihydro-15-keto PGE2	11.42	0.985	0.9	3.1	< 8	4-28
14,15-LTC4	11.48	-	-	-	-	-
LTD4	11.77	0.995	0.9	2.8	< 17	4-22
14,15-LTE4	11.82	0.993	3.6	12.1	< 22	11-30
13,14-dihydro-15-keto PGF1α	11.88	0.995	0.7	2.2	< 10	4-13
1α,1b-dihomo PGF2α	11.88	0.997	0.6	2.1	< 11	4-10
13,14-dihydro-15-keto PGD2	12.18	0.995	0.6	2.0	< 11	5-16
11-trans LTD4	12.23	0.981	0.4	1.4	< 34	7-32
PGA2	12.58	0.998	0.3	1.1	< 9	5-19
13,14-dihydro-15-keto PGD1	12.61	0.995	0.4	1.4	< 12	4-16
PGJ2	12.73	0.994	14.6	48.7	< 17	4-15
Δ12-PGJ2	12.73	0.996	0.5	1.5	< 8	7-10
LTE4	12.77	0.997	0.7	2.2	< 8	5-51
LTB5	12.90	0.983	1.5	5.0	< 29	23-31
LTC4	13.04	-	-	-	-	-
11-trans LTE4	13.18	0.987	3.2	10.6	< 43	8-24
11-trans LTC4	13.57	-	-	-	-	-

Continue Table S-3

Compound Name	Retention Time	R^2	LOD [nM]	LOQ [nM]	Precision RSD [%]	Batch-to-Batch Effect RSD [%]
12,13-DiHODE	13.62	0.953	2.7	9.0	< 12	10-19
8(S),15(S)-DiHETE	13.62	0.983	4.6	15.4	< 9	10-17
bicyclo-PGE2	13.80	0.995	1.7	5.6	< 14	4-14
17,18-DiHETE	14.01	0.990	4.7	15.5	< 11	8-26
5(S),15(S)-DiHETE	14.06	0.992	0.8	2.5	< 8	7-16
6-trans-LTB4	14.09	0.995	1.2	4.1	< 20	7-18
10(S),17(S)-DiHDoHE	14.13	0.993	0.6	1.9	< 11	5-15
15-deoxy-Δ12,14-PGD2	14.39	0.996	0.5	1.6	< 6	6-14
LTB4	14.39	0.997	1.1	3.6	< 6	4-23
14,15-DiHETE	14.49	0.993	0.8	2.7	< 13	11-22
12,13-DiHOME	14.80	0.975	0.3	1.0	< 4	3-6
9,10-DiHOME	15.18	0.981	0.5	1.8	< 8	5-9
19,20-DiHDPA	15.60	0.991	1.7	5.7	< 11	6-30
14,15-DiHETrE	15.65	0.997	0.3	1.0	< 6	2-21
12S-HHTrE	15.73	0.990	1.5	5.1	< 12	8-36
11,12-DiHETrE	16.23	0.993	0.5	1.7	< 12	6-21
9-HOTrE	16.57	0.967	0.3	1.1	< 7	5-23
8,9-DiHETrE	16.71	0.993	0.3	1.0	< 15	10-17
Hepoxilin A3	16.78	0.934	4.2	14.0	< 76	22-50
20-HETE	17.20	0.983	0.9	3.0	< 41	8-33
5(S),6(S)-DiHETE	17.20	0.992	0.4	1.4	< 20	5-22
15(S)-HEPE	17.28	0.983	0.9	3.1	< 13	11-19
5,6-DiHETrE	17.34	0.994	0.4	1.2	< 9	7-21
12(S)-HEPE	17.67	0.985	0.3	0.9	< 12	6-23
5(S)-HEPE	18.05	0.983	0.1	0.3	< 12	8-24
13-HODE	18.12	0.940	4.2	13.9	< 9	7-16
9-HODE	18.25	0.942	0.2	0.5	< 7	7-22
15-HETE	18.58	0.982	0.2	0.8	< 11	6-18
13-HpODE	18.60	0.939	3.5	11.8	< 6	23-72
17(18)-EpETE	18.60	0.994	0.5	1.7	< 17	8-22
13-KODE	18.70	0.978	0.7	2.4	< 14	8-48
9-HpODE	18.71	0.953	8.7	29.1	< 7	29-72
17-HDoHE	18.71	0.985	11.3	37.8	< 17	7-29
15-HpETE	18.93	0.985	2.3	7.7	< 24	30-75
11-HETE	18.97	0.987	0.3	0.9	< 9	4-21
15-KETE	19.03	0.988	0.8	2.8	< 13	8-34
9-KODE	19.09	0.951	3.2	10.8	< 11	10-30
14(15)-EpETE	19.09	0.988	0.8	2.6	< 19	9-23
12-HETE	19.24	0.986	0.2	0.6	< 9	4-17
8-HETE	19.25	0.990	1.4	4.7	< 8	8-21
15(S)-HETrE	19.41	0.988	0.5	1.7	< 11	7-21
12S-HpETE	19.43	0.539	-	-	< 20	51-95
9-HETE	19.47	0.963	6.5	21.7	< 12	9-25
12-KETE	19.56	-	-	-	-	-
5-HETE	19.69	0.988	0.2	0.6	< 11	14-20
19(20)-EpDPE	19.93	0.988	1.2	4.0	< 12	6-18
5(S)-HpETE	20.04	0.968	8.1	27.0	< 19	34-71
12(13)-EpOME	20.08	0.964	2.5	8.4	< 7	14-39
14(15)-EpETrE	20.19	0.986	0.9	3.1	< 27	7-21
9(10)-EpOME	20.27	0.932	0.6	1.9	< 11	10-38
16(17)-EpDPE	20.32	0.994	1.0	3.5	< 21	8-15
5-KETE	20.51	0.990	0.4	1.2	< 15	9-17
11(12)-EpETrE	20.71	0.988	0.4	1.3	< 23	16-22
8(9)-EpETrE	20.79	0.985	1.4	4.5	< 17	10-19
5(6)-EpETrE	20.95	-	-	-	-	-

Table S-4 Detected amounts [nM] of oxylipins before and 24 hours after surgery.

	Patient 1		Patient 2		Patient 3		Patient 4		Patient 5	
**Compound name**	**T0**	**T 24h**	**T0**	**T 24h**	**T0**	**T 24h**	**T0**	**T 24h**	**T0**	**T 24h**
**Arachidonic Acid**										
TXB2	<LOQ	<LOQ	<LOQ	<LOQ	<LOQ	<LOQ	<LOQ	<LOQ	<LOQ	1.5 (±0.3)
PGF2α	8.7 (±1.5)	9.3 (±2.0)	11.9 (±3.1)	15.5 (±10.1)	7.4 (±2.0)	9.9 (±2.0)	8.7 (±3.3)	6.1 (±0.4)	5.8 (±1.1)	6.7 (±0.7)
PGE2	<LOQ	0.7 (±0.2)	0.7 (±0.3)	0.8 (±0.1)	1.2 (±1.0)	<LOQ	<LOQ	1.5 (±1.0)	<LOQ	0.9 (±0.2)
11β-PGE2	<LOQ	<LOQ	<LOQ	<LOQ	<LOQ	<LOQ	<LOQ	<LOQ	<LOQ	<LOQ
13,14-dihydro PGF2α	48.9 (±3.0)	52.5 (±6.2)	65.7 (±18.8)	69.1 (±18.5)	49.2 (±5.8)	55.8 (±6.2)	50.6 (±14.1)	43.9 (±4.8)	51.5 (±4.9)	47.8 (±13.0)
14,15-DiHETrE	4.6 (±0.7)	3.6 (±0.2)	1.9 (±0.2)	2.7 (±0.3)	4.4 (±0.2)	3.1 (±0.4)	2.8 (±0.5)	1.9 (±0.2)	2.3 (±0.2)	3.4 (±0.4)
11,12-DiHETrE	3.5 (±0.4)	3.1 (±0.8)	<LOQ	2.1 (±0.9)	2.0 (±0.2)	1.9 (±0.3)	1.8 (±0.3)	<LOQ	<LOQ	2.5 (±0.9)
8,9-DiHETrE	1.6 (±0.2)	1.2 (±0.5)	<LOQ	<LOQ	<LOQ	<LOQ	<LOQ	<LOQ	<LOQ	<LOQ
5,6-DiHETrE	1.7 (±0.8)	<LOQ	<LOQ	<LOQ	<LOQ	<LOQ	<LOQ	<LOQ	<LOQ	<LOQ
15-HETE	3.4 (±1.1)	2.1 (±0.2)	1.5 (±0.5)	2.3 (±0.5)	1.9 (±0.9)	2.0 (±0.4)	1.5 (±0.3)	1.1 (±0.5)	1.3 (±0.3)	1.9 (±1.3)
11-HETE	1.6 (±0.7)	<LOQ	<LOQ	<LOQ	<LOQ	1.3 (±0.4)	<LOQ	<LOQ	<LOQ	1.4 (±0.2)
12-HETE	4.6 (±0.8)	7.3 (±2.4)	1.3 (±0.4)	9.3 (±2.4)	1.8 (±0.4)	5.6 (±0.6)	3.9 (±1.6)	8.2 (±3.2)	1.5 (±0.9)	62.9 (±8.4)
8-HETE	<LOQ	<LOQ	<LOQ	<LOQ	<LOQ	<LOQ	<LOQ	<LOQ	<LOQ	<LOQ
5-HETE	2.5 (±0.6)	1.8 (±0.2)	1.0 (±0.6)	2.4 (±0.4)	1.2 (±0.4)	3.9 (±0.6)	<LOQ	1.1 (±0.4)	1.1 (±0.3)	2.3 (±0.4)
**Linoleic Acid**										
9,12,13-TriHOME	3.6 (±0.8)	3.1 (±0.6)	2.3 (±0.3)	2.7 (±0.4)	10.7 (±1.0)	2.6 (±0.5)	18.9 (±2.1)	2.8 (±0.6)	25.2 (±2.7)	6.7 (±5.2)
9,10,13-TriHOME	2.1 (±0.6)	1.8 (±0.2)	<LOQ	<LOQ	6.5 (±0.7)	<LOQ	5.4 (±1.2)	<LOQ	4.9 (±0.2)	5.1 (±3.9)
12,13-DiHOME	14.0 (±2.4)	7.6 (±0.7)	12.6 (±0.6)	10.6 (±1.1)	63.5 (±4.0)	8.1 (±0.6)	40.9 (±2.5)	7.0 (±1.8)	39.9 (±2.9)	23.6 (±16.0)
9,10-DiHOME	7.7 (±2.5)	4.9 (±1.4)	2.6 (±0.5)	4.2 (±1.2)	80.7 (±8.4)	3.5 (±0.7)	15.3 (±2.6)	4.3 (±0.5)	20.8 (±0.9)	24.5 (±23.5)
13-HODE	29.3 (±12.6)	25.1 (±3.7)	35.7 (±3.2)	32.2 (±6.3)	104.8 (±4.9)	28.0 (±3.1)	86.5 (±3.8)	<LOQ	70.3 (±6.3)	43.5 (±18.0)
9-HODE	24.2 (±1.1)	21.2 (±2.6)	18.1 (±1.3)	26.3 (±3.3)	55.0 (±4.3)	23.0 (±2.5)	83.7 (±7.5)	13.9 (±0.8)	92.2 (±3.4)	39.3 (±13.6)
13-HpODE	200.3 (±86.1)	415.0 (±220.5)	143.7 (±96.0)	445.8 (±234.2)	513.8 (±82.8)	195.9 (±97.4)	105.5 (±38.4)	87.4 (±27.1)	80.1 (±19.5)	85.5 (±45.3)
13-KODE	9.5 (±3.1)	12.3 (±6.1)	6.3 (±1.6)	16.1 (±8.7)	19.7 (±1.5)	6.8 (±2.5)	6.2 (±0.4)	3.0 (±1.2)	5.8 (±0.3)	4.1 (±0.9)
9-HpODE	146.2 (±26.4)	227.2 (±97.0)	88.2 (±37.3)	221.7 (±141.4)	287.0 (±32.9)	142.5 (±54.4)	69.1 (±36.5)	67.5 (±44.3)	60.5 (±21.0)	72.1 (±15.5)
9-KODE	<LOQ	<LOQ	<LOQ	<LOQ	<LOQ	<LOQ	<LOQ	<LOQ	<LOQ	<LOQ
12(±13)-EpOME	11.3 (±8.2)	18.9 (±6.4)	17.1 (±4.5)	26.1 (±16.2)	26.5 (±5.5)	9.7 (±1.3)	27.2 (±4.6)	<LOQ	24.9 (±4.1)	9.1 (±2.9)
9(±10)-EpOME	7.6 (±4.2)	16.8 (±4.1)	4.2 (±1.1)	13.0 (±5.9)	16.3 (±2.9)	3.8 (±2.0)	4.6 (±1.6)	2.5 (±0.9)	5.0 (±1.1)	2.7 (±2.1)
**Dihomo-gamma-linolenic acid**										
PGF1α	<LOQ	<LOQ	5.9 (±5.5)	4.7 (±3.5)	<LOQ	<LOQ	2.7 (±1.6)	<LOQ	<LOQ	<LOQ
15(±S)-HETrE	<LOQ	<LOQ	<LOQ	<LOQ	<LOQ	<LOQ	<LOQ	<LOQ	<LOQ	<LOQ
**Alpha-linolenic acid**										
9-HOTrE	1.2 (±0.5)	<LOQ	1.5 (±0.4)	1.1 (±0.2)	3.2 (±0.7)	<LOQ	7.7 (±1.1)	<LOQ	8.4 (±0.7)	<LOQ
**Eicosapentaenoic acid**										
17,18-DiHETE	21.0 (±6.3)	17.5 (±2.8)	27.5 (±1.0)	<LOQ	27.2 (±3.3)	22.6 (±2.2)	31.4 (±9.3)	<LOQ	16.0 (±3.0)	16.5 (±0.6)
14,15-DiHETE	6.0 (±2.1)	2.9 (±1.0)	4.0 (±1.2)	2.8 (±1.2)	7.1 (±0.7)	5.6 (±2.7)	5.2 (±1.5)	3.3 (±1.1)	3.5 (±0.9)	2.9 (±1.5)
15(±S)-HEPE	<LOQ	<LOQ	<LOQ	<LOQ	<LOQ	<LOQ	<LOQ	<LOQ	<LOQ	<LOQ
12(±S)-HEPE	1.2 (±0.1)	1.3 (±0.5)	1.0 (±0.8)	2.1 (±0.8)	<LOQ	1.2 (±0.3)	1.0 (±0.4)	1.6 (±0.8)	<LOQ	4.8 (±0.8)
5(±S)-HEPE	1.8 (±0.9)	0.7 (±0.1)	1.3 (±0.1)	1.6 (±0.5)	0.8 (±0.3)	1.7 (±0.3)	0.6 (±0.2)	1.0 (±0.2)	0.4 (±0.2)	0.9 (±0.2)
**Docosahexaenoic acid**										
19,20-DiHDPA	10.3 (±1.5)	10.9 (±1.0)	9.9 (±1.4)	8.8 (±3.1)	13.3 (±0.6)	12.3 (±2.2)	10.8 (±1.0)	<LOQ	<LOQ	6.7 (±0.7)
19(±20)-EpDPE	7.2 (±2.2)	4.6 (±1.1)	<LOQ	8.6 (±7.7)	<LOQ	4.0 (±0.3)	<LOQ	<LOQ	<LOQ	4.7 (±1.4)
